# Supplementary material for: How do patients make decisions in the context of a multidisciplinary team: an ethnographic study of four head and neck cancer centres in the north of England
Source: BMJ Open. 2022 Aug 24;12(8):e061654. doi: 10.1136/bmjopen-2022-061654 (PMC9413178; doi:10.1136/bmjopen-2022-061654)
Supplement: Supplementary data [file bmjopen-2022-061654supp001.pdf]

Staff Interview Schedule

Introduce researcher and purpose of the study

Obtain consent to proceed and to record the conversation

- What do you see as the best way of making decisions in head and neck cancer?
- The MDT
  - What is it for? What is its primary aim?
  - What works well, and what works not so well?
  - How could it be improved?
  - No decision about me without me in the MDT…. Is this possible?
- Treatment decision
  - Why do you regard a treatment as the best for a patient?
  - What factors do you take into account when making treatment decisions?
- Patient involvement in decisions/shared decision making
  - What does it mean to you?
  - Do you think there are barriers to patient involvement? What are they? Why do they exist?
  - Why do clinicians struggle to involve their patients effectively?
  - Do you think we need to improve patient involvement? Why?
- Uncertainty/conflict
  - Is it a problem or is it healthy/required?
  - How should uncertainty and conflict be presented to the patient? Should they know that you don't know?
  - If there are options, how should they be communicated?
- Decision for treatment
  - What is the role of the patient in the treatment decision?
  - How much of a role should the patient have? Can patients know enough?
  - How much of a role should a patient have in the decision
    - Should we give power of vito?
    - Should you allow a patient to make a decision which is considered wrong?
    - how much should a patient know before treatment
  - How much of a role should the family have
- Palliation/prognosis
  - Do we palliate enough? Do we treat too many people radically?
  - What are the barriers to good palliation?
  - Do you think we have a range of palliative options available to us?
  - Do you have prognostic information available to you?
  - Would you use this information if it was available? How would you use it?

New patient interview 1 schedule

Introduce researcher and purpose of the study

Obtain consent to proceed and to record the conversation

The interview will cover the following broad areas:

- Previous experience of making healthcare decisions
  - What does shared decision making mean?
  - Have you had to make big healthcare decisions before?
- Experience of making this decision
  - What was your process of making the decision? Talk me though it
  - What did you draw upon?
  - What factors did you take into account?
  - Did you turn to anybody/anything for information or help?
  - How did you come to your final decision?
  - What was the MDT like?
  - Did you understand what was going on?
  - Could it be improved?
- Information given about the disease and treatment options
  - Do you think you had enough information?
  - Where do you get your information from?
  - Do you think you need more information?
  - Did you understand all the information
- Patient involvement in the decision about care
  - Should patients be involved in decisions about their care?
  - How should we involve patients?
  - Were you involved? How?
  - Would you have liked more say/less say?
  - How this made the participant feel, and further exploration
- What are your expectations of treatment?
  - Are you expecting side effects? What sort?
  - Do you have any idea of your prognosis? Do you want to know?
  - Where will you be in 6 months time?
- In the perfect world, how do you think decisions should be made?

New patient interview 2 schedule/retrospective patient interview schedule

Introduce researcher and purpose of the study

Obtain consent to proceed and to record the conversation

- Experience of the treatment
  - What has happened so far, story of the treatment
  - Correlation with expectations – worse, better
- New normal
  - Do you have a good quality of life?
  - How do you cope with swallowing?
  - How is your voice, how do you get on with communication?
- MDT and decision process
  - When you look back did you understand the treatment decision?
  - Did you understand the consequences of the decision?
  - Did you have enough information to base the decision on?
  - Should they have allowed you more or less control over the decision?
  - Do you wish the decision had been made differently?
  - Do you feel you made the right or the wrong decision?
  - Do you have any regrets?
  - How could they improve the clinic/the decision process?
  - In the perfect world, how do you think treatment decisions should be made?
- Palliation/prognosis
  - Were you ever aware of the chances of the treatment being successful?
  - Would you want to be aware?
  - Was palliation ever an option for you? Is it now?
- Information giving
  - Do you think you had enough information about treatment?
  - Did you understand what you were entering yourself into?
  - Should they give more information? Could they?
