# Supplementary material for: How do patients make decisions in the context of a multidisciplinary team: an ethnographic study of four head and neck cancer centres in the north of England
Source: BMJ Open. 2022 Aug 24;12(8):e061654. doi: 10.1136/bmjopen-2022-061654 (PMC9413178; doi:10.1136/bmjopen-2022-061654)
Supplement: Author's manuscript [file bmjopen-2022-061654.draft_revisions.pdf]

BMJ Open is committed to open peer review. As part of this commitment we make the peer review history of every article we publish publicly available.

When an article is published we post the peer reviewers' comments and the authors' responses online. We also post the versions of the paper that were used during peer review. These are the versions that the peer review comments apply to.

The versions of the paper that follow are the versions that were submitted during the peer review process. They are not the versions of record or the final published versions. They should not be cited or distributed as the published version of this manuscript.

BMJ Open is an open access journal and the full, final, typeset and author-corrected version of record of the manuscript is available on our site with no access controls, subscription charges or pay-per-view fees (http://bmjopen.bmj.com).

If you have any questions on BMJ Open's open peer review process please email info.bmjopen@bmj.com

BMJ Open

Involving patients in cancer multidisciplinary team decisions: an impossible task?

Journal:	*BMJ Open*
Manuscript ID	bmjopen-2022-061654
Article Type:	Original research
Date Submitted by the Author:	09-Feb-2022

Complete List of Authors:	Hamilton, David; Newcastle University, Population Health Sciences Institute; Newcastle Upon Tyne Hospitals NHS Foundation Trust<br>Heaven, Benjamin; Newcastle University, Population Health Sciences Institute<br>Thomson, Richard; Newcastle University, Population Health Sciences Institute<br>Wilson, Janet; University of Newcastle, Population Health Sciences Institute<br>Exley, Catherine; Newcastle University, Population Health Sciences Institute

Keywords:	Organisation of health services < HEALTH SERVICES ADMINISTRATION & MANAGEMENT, Quality in health care < HEALTH SERVICES ADMINISTRATION & MANAGEMENT, Head & neck tumours < ONCOLOGY, Head & neck surgery < OTOLARYNGOLOGY, QUALITATIVE RESEARCH

SCHOLARONE™
Manuscripts

I, the Submitting Author has the right to grant and does grant on behalf of all authors of the Work (as defined in the below author licence), an exclusive licence and/or a non-exclusive licence for contributions from authors who are: i) UK Crown employees; ii) where BMJ has agreed a CC-BY licence shall apply, and/or iii) in accordance with the terms applicable for US Federal Government officers or employees acting as part of their official duties; on a worldwide, perpetual, irrevocable, royalty-free basis to BMJ Publishing Group Ltd ("BMJ") its licensees and where the relevant Journal is co-owned by BMJ to the co-owners of the Journal, to publish the Work in this journal and any other BMJ products and to exploit all rights, as set out in our licence.

The Submitting Author accepts and understands that any supply made under these terms is made by BMJ to the Submitting Author unless you are acting as an employee on behalf of your employer or a postgraduate student of an affiliated institution which is paying any applicable article publishing charge ("APC") for Open Access articles. Where the Submitting Author wishes to make the Work available on an Open Access basis (and intends to pay the relevant APC), the terms of reuse of such Open Access shall be governed by a Creative Commons licence – details of these licences and which Creative Commons licence will apply to this Work are set out in our licence referred to above.

Other than as permitted in any relevant BMJ Author's Self Archiving Policies, I confirm this Work has not been accepted for publication elsewhere, is not being considered for publication elsewhere and does not duplicate material already published. I confirm all authors consent to publication of this Work and authorise the granting of this licence.

For peer review only

Title: Involving patients in cancer multidisciplinary team decisions: an impossible task?

DW Hamilton
Consultant Otolaryngology, Head and Neck Surgery, Honorary Senior Lecturer
Freeman Hospital, High Heaton, Newcastle upon Tyne NE7 7DN
Population Health Sciences Institute, Newcastle University, Newcastle upon Tyne, NE2 4AX

B Heaven
Formerly Newcastle University, has now left academia

R G Thomson
Emeritus Professor
Population Health Sciences Institute, Newcastle University, Newcastle upon Tyne, NE2 4AX

JA Wilson
Emerita Professor of Otolaryngology, Head and Neck Surgery
Population Health Sciences Institute, Newcastle University, Newcastle upon Tyne, NE2 4AX

C Exley
Professor of Qualitative Health Research
Population Health Sciences Institute, Newcastle University, Newcastle upon Tyne, NE2 4AX

Corresponding Author:
DW Hamilton
Freeman Hospital, High Heaton, Newcastle upon Tyne NE7 7DN
David.hamilton@ncl.ac.uk

Keywords
Decision Making, Shared
Communication
Cancer
Anthropology, Cultural
Informed consent
Autonomy

For peer review only - http://bmjopen.bmj.com/site/about/guidelines.xhtml

For peer review only

Abstract

Objectives

To describe how patients are engaged with cancer decisions in the context of multidisciplinary team (MDT) and how MDT recommendations are operationalised in the context of a shared decision.

Design

Ethnographic qualitative study

Setting

Four head and neck cancer centres in the north of England

Participants

Patients with a diagnosis of new or recurrent head and neck cancer; non-participant observation of 35 MDT meetings and 37 MDT clinics, informal interviews, and formal, semi-structured interviews with 20 patients and 9 MDT staff members

Results

MDT discussions often conclude with a firm recommendation for treatment. When delivered to a patient in clinic this recommendation is often accepted by the patient, but this response may result from the disempowered position in which they find themselves. Whilst patient behaviour may thus appear to endorse clinicians' views that a paternalistic approach is desired by patients (creating a "cycle of paternalism"), the rigidity of the MDT treatment recommendation can act as a barrier to discussion of options and the exploration of patient values.

Conclusions

The current model of MDT decision making does not support shared decision making and may actively undermine it. A model should be developed whereby the individual patient

For peer review only

perspective has more input into MDT discussions, and where decisions are made on potential treatment options rather than providing a single recommendation for discussion with the patient. Deeper consideration should be given to how the MDT incorporates the patient perspective and/or delivers its discussion of options to the patient. In order to achieve these objectives, a new model of MDT working is required.

Word count: 3732

For peer review only

For peer review only - http://bmjopen.bmj.com/site/about/guidelines.xhtml

Introduction

Multidisciplinary team (MDT) decision making is internationally mandated to support appropriate high-quality treatment of patients with cancer [1]. In the UK, MDT working was established following the Calman-Hine report [2] and improves many aspects of cancer treatment such as staging, recruitment to trials [1], adherence to treatment guidelines [3], use of effective evidence-based therapy, timeliness of care [4] and access to the allied members of the healthcare team [5]. However the practice is time consuming and expensive, costing at least £100 million a year in the UK for data preparation and the same amount again for attendance in the UK [6]. To date no MDT cost-benefit analysis has been performed. The effect on cancer survival is less clear; in head and neck cancer MDT working is reported to have a positive effect on survival [7-9], but it is difficult to determine whether survival changes over time are causally related.

MDT members report that consideration of the patient as a person in decision making as a vital part of the decision-making process. In a survey of 2054 MDT members, 95% of respondents felt that "Patient views should always inform the decision-making process" and "Patient views/preferences should be presented to the MDT by somebody who has met the patient" [10]. Omitting patient preference information has an effect on the implementation of MDT recommendations [11-13]. MDT meetings are often dominated by discussion among doctors rather than including other MDT members who may know the patient better or have a more patient-centred perspective [14-16] creating a predominance of the biomedical model of disease [17]. This means that the stated aim of many MDT members - to have the patient central to the MDT treatment discussion - is at odds with the reality of the MDT process

We have previously described that if MDT meetings are to become more patient-centred, merely introducing increasing amounts of information about the patient into the MDT is not

For peer review only - http://bmjopen.bmj.com/site/about/guidelines.xhtml

sufficient [18]. Although we know that the direct viewpoint of the patient within the MDT is lacking [19], there is to date no account of how patients engage with decisions about their treatment in the context of MDTs. This work aims to address that knowledge gap.

Methods

This qualitative study used non-participant observation and semi-structured interviews to critically examine how decisions were made in and around the MDT with a particular focus on patient centredness. All data were collected by one researcher (DWH), a head and neck surgeon. Non-participant observation enables the researcher to study participants in their natural environment, and adds value to retrospective accounts gleaned only through participant interviews [20].

Patient and Public Involvement

Two head and neck patient groups were consulted during development of the research question, study design and protocol development, but patients were not involved in data gathering and analysis.

Ethical Approval

Ethical approval was gained from the NHS Research Ethics Newcastle and North Tyneside 2 committee (reference 11/NE/0200) in September 2011 and all necessary local Research and Development governance permissions were obtained. All participant gave informed consent to be included in the study.

For peer review only - http://bmjopen.bmj.com/site/about/guidelines.xhtml

Sampling

Initial sampling aimed to recruit patients who had a treatment decision to make about their care, or where more than one treatment option was available to the patient. Concepts arising from the patient-derived data drove the subsequent data collection and analysis. A range of staff members who were part of the MDT were also recruited for interview. Purposive sampling [21] guided the sampling strategy to explore emerging concepts with data collection and analysis occurring in tandem. Thus, further sampling was guided by the emerging analysis [22] and continued until a state of theoretical sufficiency [23] was achieved.

Observations

Non-participant observations of 35 MDT meetings and 37 MDT outpatient clinics were conducted. Patient with a diagnosis of new or recurrent head and neck cancer whose treatment options were being discussed in the MDT were included. They were excluded if they did not understand written or spoken English, or they did not have the capacity to consent. The MDT meetings and clinics were all audio-recorded and transcribed verbatim. Detailed field notes were also made at the time of observation, then transcribed immediately afterwards.

Interviews

Semi-structured interviews were conducted with patients and staff. The development of the interview guide was iterative; as data collection continued, the content of the guide evolved in order to explore emerging themes. Informal interviews with staff members of the MDT also took place and were incorporated into written field notes. Pseudonyms are used for reporting data throughout to protect the anonymity of respondents.

For peer review only

Analysis

The data were analysed by one researcher (DWH) and emerging analyses were discussed with CE and BH, following principles of constructivist grounded theory [21]. All data sources (MDT meeting, clinic, informal and formal interviews) were analysed using the same coding framework. Line by line coding produced an initial coding framework: the emerging analysis was used during axial coding to guide further sampling and further development of the coding framework; when the coding framework was altered, all transcripts were re-coded. The codes used were conceptual, rather than descriptive, and labels were derived completely from the data, not pre-determined. The coding was organised using the NVivo computer package. Emerging findings (and 'memos') were discussed in the research team to develop the data analysis and guide subsequent analysis and data sampling

Results

The research was conducted in four head and neck cancer (HNC) centres in the north east of England. In all centres, the MDT meeting took place without the patient present; following this, one or more members of the MDT met with the patient in clinic.

MDT recommendation for "best treatment"

The MDT meeting discussion often tends towards debate on which treatment is "best" for a patient amongst the available options. In the following interview extract, a maxillofacial surgeon describes his view of the aim of the MDT discussion:

> [The team] need to leave the MDT [meeting] with the treatment options ….prioritised. So a rank order of [the] best treatment clinically – slightly irrespective of the patient's wishes. From a clinical point of view to try and get best outcome, this would be our first, this would be our second, this would be third and fourth and fifth. Then you discuss it with the patient and say, "This is what we think."

For peer review only - http://bmjopen.bmj.com/site/about/guidelines.xhtml

In this data extract, the surgeon clearly states his view that the aim of the MDT discussion is to decide the "clinically" best treatment for the patient and even goes as far to say that this could be "irrespective" of the patient's wishes. Teams frequently conclude their discussion of treatment options in the MDT meeting with an agreement for the recommendation which is to be delivered to the patient:

> Mr Black (ENT surgeon): I have a database of the [laser resections] I have done …. tonsil and soft palate tumours, and it's just….it's something we need to take notice of
>
> Mr Red (ENT surgeon): Yeah, I think we'll need to, we'll have to discuss it another time or we'll take up the whole morning on one case. But, I think there are arguments for and against...
>
> Dr Orange (oncologist): I would suggest he has radiotherapy, because he will have a slightly better functional outcome, and he's 80 and …because of his age, and because of the possibly better function….would you Dr Yellow?
>
> Dr Yellow (oncologist): Yes
>
> Mr Red: I think there is a consensus view of the MDT, would be for radiotherapy
>
> MR Black: OK
>
> (Observation, MDT meeting)

Although, during this discussion, options of radiotherapy and laser were available to the patient, the position of the MDT meeting was to provide a recommendation for radiotherapy. This recommendation for "best treatment" is often conveyed to the patient on its own or in preference to other options (we have described this further in our previous paper [18])

The "cycle of paternalism"

Anxious patients, faced with complex decisions in the setting of a potentially life-limiting disease often turn to their clinicians for guidance. This, in turn, leads to patients endorsing the paternalistic approach as they are given little or no information about the available treatment choices and therefore tend to delegate responsibility of the decision to the clinician:

For peer review only - http://bmjopen.bmj.com/site/about/guidelines.xhtml

> Pt: You know, they're the doctors, they're the professional people. And I'm just Joe Bloggs off the street. ...For a lot of years, I was a steel erector. I wouldn't expect you as a doctor coming along and doing what I could do. Do I?
>
> Interviewer: You feel a decision should be the doctor's decision?
>
> Pt: Oh, definitely, without a doubt. It's got to be the doctor's decision. How could I make a decision like that?

Here, the patient delegates the decision to the MDT without question; he is allowing the MDT's assessment of 'best' treatment to act as the sole basis for a treatment decision. If decision delegation is accepted as the method by which MDTs convey and make decisions, a paternalistic decision making process results. In this model, the patient accepts that the MDT's assessment of 'best' (and hence the treatment recommendation) is appropriate. It creates a "cycle of paternalism" with grateful patients accepting firm recommendations from clinicians and clinicians reassured that they are doing their best for their patients.

Delivery of the MDT treatment recommendation

Firm MDT recommendations can sometimes place the clinician in a difficult position when discussing options. The following extract is the clinic appointment for Vincent Lowry (the MDT meeting extract was included above). Here Mr Black (who favoured laser in the MDT meeting, referred to here as "surgery") was delivering the MDT recommendation for radiotherapy to the patient:

> Mr Black: After a lot of discussion, the consensus…. would be to give you radiation therapy…. that was what we jointly decided. And we think with that treatment there is a very good chance of controlling your disease completely….
>
> Pt: Well, I'll do as you say
>
> Daughter: So there's no other operation, it would just be radiotherapy?
>
> Mr Black: We discussed this at length at the meeting…. and the majority of people… felt that to be frank, except for me, felt that radiation would be the way forward. And…. that's what we are offering to you as first line

For peer review only - http://bmjopen.bmj.com/site/about/guidelines.xhtml

treatment. Unless you have any reservations, then we can think about other options.

Pt: I'll do as you say….

Daughter: Right. So he would have to come into hospital every day? He's a really bad traveller…

Pt: You know when I come here I get all tensed up and travelling….

Mr Black: Really? Is it likely you may then stop the treatment midway for whatever reason, because that would backfire very badly.

Pt: I wouldn't do that

Mr Black: I know you asked about the surgical option. I promised people I wouldn't say anything. But it is feasible to take it out surgically, and there is an option available, but the consensus at the MDT was to go ahead with radiation. Unless, as a family or yourself very strongly object to it and feel that you can't go ahead with that, then of course the surgical options is always there. But as a group we felt that the best way forward was to offer you radiation

Pt: Well. I'll go with you

(Observation, MDT Clinic)

The final treatment decision was to deliver radiation, but the interaction above reveals the challenges of being tasked by the MDT to give a single recommendation when it is used in a decision discussion with a patient. Once new information was gleaned from the patient in clinic (being a "really bad traveller") Mr Black struggled with how to deal with the recommendations: was it a rule to be followed? The data presented also show that the level of patient involvement is often to either accept or "object" to the MDT recommendation. Not only is the patient given no basis for these objections, limiting patient involvement in such a way does not constitute shared decision-making. Here, the rigidity of the treatment recommendation acted as a barrier to an open discussion about the treatment options available to the patient and thus inhibited shared decision making.

For peer review only - http://bmjopen.bmj.com/site/about/guidelines.xhtml

For peer review only

Patient engagement with MDT recommendations

Modern clinical practice cannot assume that the sole role of the patient is the acceptance of a single firm treatment recommendations. John Winton was a 61 year old patient with an advanced cancer of his larynx. In the MDT it was decided that surgery would provide him with the best chance of survival but would remove his voice box. Radiotherapy is available, carries a lower chance of cure, but allows him to retain his voice box. The following data are from his clinic appointment:

> Mr Black (ENT surgeon): This tumour in your throat is a fairly big tumour, and it's spread to the neck as well. We believe that there are two possible ways that we can manage this. At some parts of the scan, there is evidence that the tumour may have gone into the Adam's apple cartilage…. If that is the case, surgery would be the only option to get rid of the tumour. But surgery would involve you losing your voicebox, losing part of the swallowing passage, you would need a big neck operation….Once we do the surgery, your speech will be different, you won't be speaking the same. You will have a hole in the centre of your neck, a tracheostomy
>
> Pt: Nah, nah [shakes head]
>
> Mr Black: You wouldn't fancy that?
>
> Pt: No
>
> Mr Black: That's the surgical option. On the other side is the option of radiation therapy
>
> Pt: I would rather take a chance with that
>
> (Observation, MDT Clinic)

Here, and throughout the course of this consultation, the patient made a decision to reject surgery, which reduces his length of survival from his cancer in order to preserve his voice box. He was adamant he did not want a complete removal of the voice box and part of the throat (pharygolaryngectomy) and the decision was eventually made to use radiotherapy. However, in the subsequent interview, Mr Winton discussed the rationale for his decision:

> Pt: Well you see my mother died of cancer… my father died of cancer, and I've seen the way cancer works. I'm not being cheeky…. once they cut you

For peer review only - http://bmjopen.bmj.com/site/about/guidelines.xhtml

> open, it's like your letting fresh air into a bulb, it then just spreads, and they stitch you back up again and "We've cured it", right, for how long? And then it comes back again…
>
> Interviewer: And what's important to you when you're making that decision?
>
> Pt: Surviving as long as I can…, I mean if you get the year, 18 months it's better than getting two weeks isn't it?

His consultation, which was limited in exploring what mattered to him, leads to a decision that is potentially at odds with his aspirations revealed above. His aim of treatment (survival) is not matched by the actual treatment decision (radiotherapy). This patient perspective could not be incorporated into the previous MDT discussion (which happened before the clinic appointment), but equally the subsequent clinic appointment did not explore his preferences and what underpinned them, risking a treatment decision at odds with his preferences and values. If Mr Black had explored the options for treatment with the patient more, this mismatch of treatment preferences and values could have been identified, and perhaps deconstructed. Such information about values and preferences is essential to good shared decision making, however very difficult to incorporate into the MDT decision making structure.

Discussion

The outcome of an MDT discussion is frequently a "best" or preferred treatment recommendation to act as the basis of a treatment decision to be delivered in the MDT clinic. Often patients accept this recommendation in the clinic (perhaps precisely because it is presented as the "best" treatment). However, this acquiescence may be due to the disempowered position in which patients find themselves as they confront a terrifying diagnosis and a myriad of complex decision options. In turn, clinicians often view the acceptance of an MDT recommendation as delegation of the decision by the patient to the

For peer review only - http://bmjopen.bmj.com/site/about/guidelines.xhtml

clinician, an assumption which can promulgate a ‘cycle of paternalism’, where anxious patients have little real choice other than to accept the clear guidance offered by the expert team. Arguably, this paternalistic model of decision making is the inevitable result of the current MDT structure of working which does not adequately include the patient view, values or preferences which are key to a shared treatment decision.

The rigidity of the MDT recommendation can act as a barrier to an open discussion of the available options. If the patient role is limited to either acceptance or refusal of a single recommendation, true engagement is impossible. A truncated discussion of a single MDT recommendation for treatment prohibits shared decision-making using the “three talk model” ,as central to this model is a discussion of the options for treatment. A shared decision-making consultation allows the patient and clinician to explore the risks, benefits and consequences of a treatment alternatives; a move from initial to informed preferences; and exploration of patient values to reach a shared decision [24].

The structure of MDT working has not significantly changed since its inception in 1996. NHS patients do not routinely attend their MDT meetings, modern cancer care mandates that all patients are discussed in this setting[25] and interventions to increase the number of patients discussed in an MDT are still sought after[26].

The MDT recommendation

If the MDT meeting and clinic follow a paternalistic pathway, the way in which their recommendation is used is clear: it is delivered to the patient with an assumption that it will be accepted. Outwith the MDT decision process, a treatment recommendation from an individual clinician can be modified depending on the ongoing interaction with the patient and the preferences expressed. An MDT recommendation, on the other hand, is problematic for MDT members who attempt to combine it with the values or preferences of the patient. Is

For peer review only - http://bmjopen.bmj.com/site/about/guidelines.xhtml

it set in stone, an obligatory 'best' which must be adhered to? If the patient disagrees with the recommendation, what action should the MDT member take? In this way, MDT recommendations are inflexible, especially in the light of new information from the patient which was not clear or known in the MDT meeting. In other words, information about values and preferences are vital to a shared decision but difficult to incorporate into the MDT decision making structure. As we have previously described [18], MDTs often build the "evidential patient" in the MDT meeting discussion. This may include information about a patient's values and preferences, but these are impossible to incorporate into a meeting discussion without the patient present and without making assumptions about the patient.

Modernising MDT decision-making

If we are to modernise the MDT decision making structure to improve patient involvement, the role of the MDT discussion and the structure of the clinic must recognise that patients often "distribute" decisions. Rapley [27] describes how patients demonstrate a 'relational autonomy' by distributing their decision amongst people, encounters, places and information sources. Promoting relational autonomy means that involving patients in decisions requires more than presenting options and awaiting a verdict, instead emphasising the importance of the interaction with the clinician, encouraging questions, correcting misunderstanding, constructing preferences and allowing disagreement [28]. Indeed, the MDT decision-making structure gives ample opportunity for MDT members to distribute their decision amongst colleagues, but does not afford the same opportunity to patients

If the patient is to be a true participant in shared decision making, an alternative model of MDT decision making is required. Some teams may explore the idea of a patient attending their own MDT meeting: this idea is popular amongst patient advocates [29], but not clinicians [10 29]. There is little data documenting the patient experience of involvement in MDTs, as it is

For peer review only - http://bmjopen.bmj.com/site/about/guidelines.xhtml

rarely routine practice. Small studies have concluded that patients attending their own MDT allows for better information giving but not necessarily improved involvement in decision making [30 31]. MDT members often feel that patients attending their own meeting would inhibit the discussion and cause patient anxiety [29]; relationships within the MDT are often longstanding with pre existing hierarchies which can present barriers to new user integration[32]. However, the concept of a patient prepared with information about the disease and MDT team structure, with a supporter or team member as advocate, attending a sympathetic MDT, is worth further exploration.

Of key importance is that the MDT meeting is not a discussion of which option is 'best' for a particular patient, but should instead aim to determine which valid treatment options are available. In particular, palliative options (or options of 'doing nothing') are often inadequately explored [33]. Clinic structures should be flexible to allow patients to distribute their decision-making amongst information sources and people. The patient may be enabled to come to the initial consultation more informed and prepared for the discussion. There may be a role for pre MDT clinic with the patient meeting a surgeon, oncologist or specialist nurse, or a post MDT clinic to convey options and explore values and preferences, maybe with more than one clinician. The MDT meeting may take place in a small 'combined clinic' setting around the interaction with the patient. The MDT members provide support, resources and personnel to discuss the treatment options, communicate the risk and uncertainty, elicit values and explore them; a decision aid may support this work [34]. The team may consider providing an individual who is independent of the clinical team to act as a decision coach or navigator [35]. MDT members should be encouraged to update their training in supporting patients in shared decision making, consent and communication.

For peer review only - http://bmjopen.bmj.com/site/about/guidelines.xhtml

It is time for the development and design of alternative models of team decision making which have a central role for the patient. Further work to develop new model of delivering team decision-making would be multifactorial, incorporating the development of the structure of the MDT meeting and clinic, support and training for MDT members and patients and the development of tools to be used in combination with team decisions. Qualitative approaches should explore stakeholders' views of intervention components, which should be co-designed with patients. Evaluation of such interventions requires novel trial design, comparing methods of decision making and evaluating decision quality. MDT decision making is now ubiquitous and therefore the urgent need of reform to meet the principles of shared decision making should be a priority for clinical teams and cancer researchers.

For peer review only

Author Contributions

DW Hamilton
Protocol development, ethical approval, data gathering, data analysis, manuscript preparation

B Heaven
Protocol development, ethical approval, data analysis, manuscript preparation

R G Thomson
Protocol development, data analysis, manuscript preparation

JA Wilson
Protocol development, data analysis, manuscript preparation

C Exley
Protocol development, ethical approval, data analysis, manuscript preparation

Competing interests

All authors have completed the Unified Competing Interest form at www.icmje.org/coi_disclosure.pdf (available on request from the corresponding author) and declare that DWH, BH, RT, JAW and CE have no relationships with any companies that might have an interest in the submitted work in the previous 3 years; and DWH, BH, RT, JAW and CE have no non-financial interests that may be relevant to the submitted work

For peer review only - http://bmjopen.bmj.com/site/about/guidelines.xhtml

Funding

This study represents independent research funded by the National Institute of Health Research. The views expressed are those of the author(s) and not necessarily those of the National Health Service, the National Institute of Health Research or the Department of Health

Data sharing statement

No additional data available

Strengths and limitations of this study

- This ethnographic study provides an in-depth analysis of the complexities of patient involvement and interaction with MDT decision making
- The methods (direct observation and semi structured interviews) allow a rich, data-driven analysis of a complex decision-making environment
- Head and neck cancer involves the trade off of function for survival and is thus a useful model when exploring complex decision making
- All data involve patients with one cancer area in a small number of centres; whilst the MDT model predominates there are alternative structures of team decision making
- Some of the challenges described in this work may not be encountered by other cancers and other centres using the MDT model

References

1. Licitra L, Keilholz U, Tahara M, et al. Evaluation of the benefit and use of multidisciplinary teams in the treatment of head and neck cancer. *Oral Oncol* 2016;59:73-9. doi: 10.1016/j.oraloncology.2016.06.002

2. Calman KC, Hine D. A Policy Framework for Commissioning Cancer Services In: Health Do, ed., 1995.

For peer review only - http://bmjopen.bmj.com/site/about/guidelines.xhtml

3. Kelly SL, Jackson JE, Hickey BE, et al. Multidisciplinary clinic care improves adherence to best practice in head and neck cancer. *American journal of otolaryngology* 2013;34(1):57-60. doi: 10.1016/j.amjoto.2012.08.010

4. Patil RD, Meinzen-Derr JK, Hendricks BL, et al. Improving access and timeliness of care for veterans with head and neck squamous cell carcinoma: A multidisciplinary team's approach. *Laryngoscope* 2016;126(3):627-31. doi: 10.1002/lary.25528

5. Starmer H, Sanguineti G, Marur S, et al. Multidisciplinary head and neck cancer clinic and adherence with speech pathology. *Laryngoscope* 2011;121(10):2131-5. doi: 10.1002/lary.21746

6. Taylor C, Munro AJ, Glynne-Jones R, et al. Multidisciplinary team working in cancer: what is the evidence? *Bmj* 2010;340:c951. doi: 10.1136/bmj.c951

7. Wang YH, Kung PT, Tsai WC, et al. Effects of multidisciplinary care on the survival of patients with oral cavity cancer in Taiwan. *Oral Oncol* 2012;48(9):803-10. doi: 10.1016/j.oraloncology.2012.03.023

8. Birchall M, Bailey D, King P, et al. Effect of process standards on survival of patients with head and neck cancer in the south and west of England. *British journal of cancer* 2004;91(8):1477-81. doi: 10.1038/sj.bjc.6602118

9. Friedland PL, Bozic B, Dewar J, et al. Impact of multidisciplinary team management in head and neck cancer patients. *British journal of cancer* 2011;104(8):1246-8. doi: 10.1038/bjc.2011.92

10. NCAT. Multidisciplinary team members' views about MDT working: results from a survey commissioned by the National Cancer Action Team. London Kings College, 2009.

11. Rajan S, Foreman J, Wallis MG, et al. Multidisciplinary decisions in breast cancer: does the patient receive what the team has recommended? *British journal of cancer* 2013;108(12):2442-7. doi: 10.1038/bjc.2013.267

12. Blazeby JM, Wilson L, Metcalfe C, et al. Analysis of clinical decision-making in multi-disciplinary cancer teams. *Annals of oncology : official journal of the European Society for Medical Oncology / ESMO* 2006;17(3):457-60. doi: 10.1093/annonc/mdj102

13. Wood JJ, Metcalfe C, Paes A, et al. An evaluation of treatment decisions at a colorectal cancer multi-disciplinary team. *Colorectal disease : the official journal of the Association of Coloproctology of Great Britain and Ireland* 2008;10(8):769-72. doi: 10.1111/j.1463-1318.2007.01464.x

14. Taylor C, Atkins L, Richardson A, et al. Measuring the quality of MDT working: an observational approach. *BMC cancer* 2012;12:202. doi: 10.1186/1471-2407-12-202

15. Rowlands S, Callen J. A qualitative analysis of communication between members of a hospital-based multidisciplinary lung cancer team. *Eur J Cancer Care* 2013;22(1):20-31. doi: 10.1111/ecc.12004

For peer review only

For peer review only - http://bmjopen.bmj.com/site/about/guidelines.xhtml

16. Lanceley A, Savage J, Menon U, et al. Influences on multidisciplinary team decision-making. *Int J Gynecol Cancer* 2008;18(2):215-22. doi: DOI 10.1111/j.1525-1438.2007.00991.x

17. Kidger J, Murdoch J, Donovan JL, et al. Clinical decision-making in a multidisciplinary gynaecological cancer team: a qualitative study. *Bjog-Int J Obstet Gy* 2009;116(4):511-17. doi: DOI 10.1111/j.1471-0528.2008.02066.x

18. Hamilton DW, Heaven B, Thomson RG, et al. Multidisciplinary team decision-making in cancer and the absent patient: a qualitative study. *BMJ Open* 2016;6(7):e012559. doi: 10.1136/bmjopen-2016-012559 [published Online First: 2016/07/23]

19. Taylor C, Finnegan-John J, Green JS. "No decision about me without me" in the context of cancer multidisciplinary team meetings: a qualitative interview study. *BMC health services research* 2014;14:488. doi: 10.1186/s12913-014-0488-2

20. Strauss AL. Continual permutations of action. New York: Aldine de Gruyter 1993.

21. Charmaz K, editor. *Constructing Grounded Theory. A Practical Guide Through Qualitative Analysis*. London: SAGE, 2006.

22. Strauss AL, Corbin JM. Basics of qualitative research : grounded theory procedures and techniques. Newbury Park, Calif.: Sage Publications 1990.

23. Dey I. Grounding grounded theory : guidelines for qualitative inquiry. San Diego: Academic Press 1999.

24. Elwyn G, Durand MA, Song J, et al. A three-talk model for shared decision making: multistage consultation process. *Bmj* 2017;359:j4891. doi: 10.1136/bmj.j4891 [published Online First: 2017/11/08]

25. NICE. Improving Outcomes in Head and Neck Cancer - the Manual. London, 2004.

26. Maharaj AD, Evans SM, Zalcberg JR, et al. Barriers and enablers to the implementation of multidisciplinary team meetings: a qualitative study using the theoretical domains framework. *BMJ quality & safety* 2021;30(10):792-803. doi: 10.1136/bmjqs-2020-011793 [published Online First: 2020/11/29]

27. Rapley T. Distributed decision making: the anatomy of decisions-in-action. *Sociol Health Illn* 2008;30(3):429-44. doi: SHIL1064 [pii]

10.1111/j.1467-9566.2007.01064.x [published Online First: 2008/01/16]

28. Entwistle VA, Carter SM, Cribb A, et al. Supporting patient autonomy: the importance of clinician-patient relationships. *J Gen Intern Med* 2010;25(7):741-5. doi: 10.1007/s11606-010-1292-2

29. Butow P, Harrison JD, Choy ET, et al. Health professional and consumer views on involving breast cancer patients in the multidisciplinary discussion of their disease and treatment plan. *Cancer* 2007;110(9):1937-44. doi: 10.1002/cncr.23007

For peer review only - http://bmjopen.bmj.com/site/about/guidelines.xhtml

30. Bellardita L, Donegani S, Spatuzzi AL, et al. Multidisciplinary Versus One-on-One Setting: A Qualitative Study of Clinicians' Perceptions of Their Relationship With Patients With Prostate Cancer. *Journal of oncology practice / American Society of Clinical Oncology* 2011;7(1):e1-5. doi: 10.1200/JOP.2010.000020

31. Choy ET, Chiu A, Butow P, et al. A pilot study to evaluate the impact of involving breast cancer patients in the multidisciplinary discussion of their disease and treatment plan. *Breast* 2007;16(2):178-89. doi: 10.1016/j.breast.2006.10.002

32. Martin GP, Finn R. Patients as team members: opportunities, challenges and paradoxes of including patients in multi-professional healthcare teams. *Sociol Health Illn* 2011;33(7):1050-65. doi: 10.1111/j.1467-9566.2011.01356.x

33. Nelson A, Longo M, Byrne A, et al. Chemotherapy decision-making in advanced lung cancer: a prospective qualitative study. *BMJ Support Palliat Care* 2020 doi: 10.1136/bmjspcare-2020-002395 [published Online First: 2020/08/23]

34. Stacey D, Legare F, Lewis K, et al. Decision aids for people facing health treatment or screening decisions. *Cochrane Db Syst Rev* 2017(4) doi: ARTN CD001431

10.1002/14651858.CD001431.pub5

35. Stacey D, Murray MA, Legare F, et al. Decision coaching to support shared decision making: a framework, evidence, and implications for nursing practice, education, and policy. *Worldviews Evid Based Nurs* 2008;5(1):25-35. doi: WVN108 [pii]

10.1111/j.1741-6787.2007.00108.x [published Online First: 2008/02/13]

For peer review only

For peer review only - http://bmjopen.bmj.com/site/about/guidelines.xhtml

Standards for Reporting Qualitative Research (SRQR) Checklist

1) **Title:** Page 1

2) **Abstract**: Page 2-3

4) **Purpose or research question:** page 4-5

5) **Qualitative approach and research paradigm:** Pages 5-7

6) **Researcher characteristics and reflexivity**: Page 5

7) **Context**: page 6 and page 7

8) **Sampling strategy**: page 7

9) **Ethical issues pertaining to human subjects**: page 5

10) **Data collection methods**: page 6

11) **Data collection instruments and technologies**: page 6

12) **Units of study**: page 6

13) **Data processing:** page 6-7

14) **Data analysis:** page 7

15) **Techniques to enhance trustworthiness:** page 7

16) **Synthesis and interpretation**: page 7-12

17) **Links to empirical data:** Page 7-12

18) **Integration with prior work, implications, transferability and contributions to the field:** page 12-16

20) **Conflict of interest**: page 16

21) **Funding**: page 17

For peer review only

BMJ Open

How do patients make decisions in the context of a multidisciplinary team: an ethnographic study of four head and neck cancer centres

Journal:	*BMJ Open*
Manuscript ID	bmjopen-2022-061654.R1
Article Type:	Original research
Date Submitted by the Author:	30-May-2022
Complete List of Authors:	Hamilton, David; Newcastle University, Population Health Sciences Institute; Newcastle Upon Tyne Hospitals NHS Foundation Trust<br>Heaven, Benjamin; Newcastle University, Population Health Sciences Institute<br>Thomson, Richard; Newcastle University, Population Health Sciences Institute<br>Wilson, Janet; University of Newcastle, Population Health Sciences Institute<br>Exley, Catherine; Newcastle University, Population Health Sciences Institute
**Primary Subject Heading**:	Patient-centred medicine
Secondary Subject Heading:	Communication, Ear, nose and throat/otolaryngology, Health services research, Qualitative research
Keywords:	Organisation of health services < HEALTH SERVICES ADMINISTRATION & MANAGEMENT, Quality in health care < HEALTH SERVICES ADMINISTRATION & MANAGEMENT, Head & neck tumours < ONCOLOGY, Head & neck surgery < OTOLARYNGOLOGY, QUALITATIVE RESEARCH

SCHOLARONE™
Manuscripts

I, the Submitting Author has the right to grant and does grant on behalf of all authors of the Work (as defined in the below author licence), an exclusive licence and/or a non-exclusive licence for contributions from authors who are: i) UK Crown employees; ii) where BMJ has agreed a CC-BY licence shall apply, and/or iii) in accordance with the terms applicable for US Federal Government officers or employees acting as part of their official duties; on a worldwide, perpetual, irrevocable, royalty-free basis to BMJ Publishing Group Ltd ("BMJ") its licensees and where the relevant Journal is co-owned by BMJ to the co-owners of the Journal, to publish the Work in this journal and any other BMJ products and to exploit all rights, as set out in our licence.

The Submitting Author accepts and understands that any supply made under these terms is made by BMJ to the Submitting Author unless you are acting as an employee on behalf of your employer or a postgraduate student of an affiliated institution which is paying any applicable article publishing charge ("APC") for Open Access articles. Where the Submitting Author wishes to make the Work available on an Open Access basis (and intends to pay the relevant APC), the terms of reuse of such Open Access shall be governed by a Creative Commons licence – details of these licences and which Creative Commons licence will apply to this Work are set out in our licence referred to above.

Other than as permitted in any relevant BMJ Author's Self Archiving Policies, I confirm this Work has not been accepted for publication elsewhere, is not being considered for publication elsewhere and does not duplicate material already published. I confirm all authors consent to publication of this Work and authorise the granting of this licence.

For peer review only

Title: How do patients make decisions in the context of a multidisciplinary team: an ethnographic study of four head and neck cancer centres

DW Hamilton
Consultant Otolaryngology, Head and Neck Surgery, Honorary Senior Lecturer
Freeman Hospital, High Heaton, Newcastle upon Tyne NE7 7DN
Population Health Sciences Institute, Newcastle University, Newcastle upon Tyne, NE2 4AX

B Heaven
Formerly Newcastle University, has now left academia

R G Thomson
Emeritus Professor
Population Health Sciences Institute, Newcastle University, Newcastle upon Tyne, NE2 4AX

JA Wilson
Emerita Professor of Otolaryngology, Head and Neck Surgery
Population Health Sciences Institute, Newcastle University, Newcastle upon Tyne, NE2 4AX

C Exley
Professor of Qualitative Health Research
Population Health Sciences Institute, Newcastle University, Newcastle upon Tyne, NE2 4AX

Corresponding Author:
DW Hamilton
Freeman Hospital, High Heaton, Newcastle upon Tyne NE7 7DN
David.hamilton@ncl.ac.uk

Keywords
Decision Making, Shared
Communication
Cancer
Anthropology, Cultural
Informed consent
Autonomy

For peer review only

Abstract

Objectives

To describe how patients are engaged with cancer decisions in the context of multidisciplinary team (MDT) and how MDT recommendations are operationalised in the context of a shared decision.

Design

Ethnographic qualitative study

Setting

Three head and neck cancer centres in the north of England

Participants

Patients with a diagnosis of new or recurrent head and neck cancer; non-participant observation of 35 MDT meetings and 37 MDT clinics, informal interviews, and formal, semi-structured interviews with 20 patients and 9 MDT staff members

Methods

Ethnographic methods including non-participant observation of MDT meetings and clinic appointments, informal interviews, field notes and formal semi-structured interviews with patients and MDT members

Results

MDT discussions often conclude with a firm recommendation for treatment. When delivered to a patient in clinic this recommendation is often accepted by the patient, but this response may result from the disempowered position in which they find themselves. Whilst patient behaviour may thus appear to endorse clinicians' views that a paternalistic approach is desired by patients (creating a "cycle of paternalism"), the rigidity of the MDT treatment

For peer review only - http://bmjopen.bmj.com/site/about/guidelines.xhtml

recommendation can act as a barrier to discussion of options and the exploration of patient values.

Conclusions

The current model of MDT decision making does not support shared decision making and may actively undermine it. A model should be developed whereby the individual patient perspective has more input into MDT discussions, and where decisions are made on potential treatment options rather than providing a single recommendation for discussion with the patient. Deeper consideration should be given to how the MDT incorporates the patient perspective and/or delivers its discussion of options to the patient. In order to achieve these objectives, a new model of MDT working is required.

Word count: 3732

Strengths and limitations of this study

- This ethnographic study provides an in-depth analysis of the complexities of patient involvement and interaction with MDT decision making
- The methods (direct observation and semi structured interviews) allow a rich, data-driven analysis of a complex decision-making environment
- Head and neck cancer involves the trade off of function for survival and is thus a useful model when exploring complex decision making
- All data involve patients with one cancer area in a small number of centres; whilst the MDT model predominates there are alternative structures of team decision making Internationally, there are multiple models of MDT decision making. Although the structure discussed here predominates in the UK, the issues faced will not be applicable to all teams

For peer review only - http://bmjopen.bmj.com/site/about/guidelines.xhtml

Introduction

Multidisciplinary team (MDT) decision making is internationally mandated to support appropriate high-quality treatment of patients with cancer [1]. In the UK, MDT working was established following the Calman-Hine report [2] and improves many aspects of cancer treatment such as staging, recruitment to trials [1], adherence to treatment guidelines [3], use of effective evidence-based therapy, timeliness of care [4] and access to the allied members of the healthcare team [5]. However the practice is time consuming and expensive, costing at least £100 million a year in the UK for data preparation and the same amount again for attendance in the UK [6]. To date no MDT cost-benefit analysis has been performed. The effect on cancer survival is less clear; in head and neck cancer MDT working is reported to have a positive effect on survival [7-9], but it is difficult to determine whether survival changes over time are causally related.

MDT members report that consideration of the patient as a person in decision making as a vital part of the decision-making process. In a survey of 2054 MDT members, 95% of respondents felt that "Patient views should always inform the decision-making process" and "Patient views/preferences should be presented to the MDT by somebody who has met the patient" [10]. Omitting patient preference information has an effect on the implementation of MDT recommendations [11-13]. MDT meetings are often dominated by discussion among doctors rather than including other MDT members who may know the patient better or have a more patient-centred perspective [14-17] creating a predominance of the biomedical model of disease [18-20]. This means that the stated aim of many MDT members - to have the patient central to the MDT treatment discussion - is at odds with the reality of the MDT process

We have previously described that if MDT meetings are to become more patient-centred, merely introducing increasing amounts of information about the patient into the MDT is not

For peer review only - http://bmjopen.bmj.com/site/about/guidelines.xhtml

sufficient [21]. Although we know that the direct viewpoint of the patient within the MDT is lacking [22], there is to date no account of how patients engage with decisions about their treatment in the context of MDTs. This work aims to explore the experience of making decisions in the context of an MDT, with a particular emphasis on the patient experience of the decision process.

Methods

This qualitative study used non-participant observation and semi-structured interviews to critically examine how decisions were made in and around the MDT with a particular focus on patient centredness. All data were collected by one researcher (DWH), a head and neck surgeon. Non-participant observation enables the researcher to study participants in their natural environment, and adds value to retrospective accounts gleaned only through participant interviews [23].

Patient and Public Involvement

Two head and neck patient groups were consulted during development of the research question, study design and protocol development, but patients were not involved in data gathering and analysis.

Ethical Approval

Ethical approval was gained from the NHS Research Ethics Newcastle and North Tyneside 2 committee (reference 11/NE/0200) in September 2011 and all necessary local Research and Development governance permissions were obtained. All participant gave informed consent to be included in the study.

For peer review only - http://bmjopen.bmj.com/site/about/guidelines.xhtml

Sampling

Initial sampling aimed to recruit patients who had a treatment decision to make about their care, or where more than one treatment option was available to the patient. Concepts arising from the patient-derived data drove the subsequent data collection and analysis. A range of staff members who were part of the MDT were also recruited for interview. Purposive sampling [24] guided the sampling strategy to explore emerging concepts with data collection and analysis occurring in tandem. Cases were included which would test the concepts and themes which were emerging. For example, in the early cases, palliative options were often not discussed or offered in the clinic, and so patients were included who had options for treatment, one of which was palliative, were included. Concepts explored through sampling also included uncertainty, assessment of best and trust. Thus, further sampling was guided by the emerging analysis [25] and continued until a state of theoretical sufficiency [26] was achieved. This means that data collection ceases when sufficient or adequate depth of understanding has been reached; this allows for a greater number and breadth of concepts to be explored in this complex setting using multiple data collection techniques

Observations

Non-participant observations of 35 MDT meetings and 37 MDT outpatient clinics were conducted. Patient with a diagnosis of new or recurrent head and neck cancer whose treatment options were being discussed in the MDT were included. They were excluded if they did not understand written or spoken English, or they did not have the capacity to consent. The MDT meetings and clinics were all audio-recorded and transcribed verbatim. Detailed field notes were also made at the time of observation, then transcribed immediately afterwards.

For peer review only - http://bmjopen.bmj.com/site/about/guidelines.xhtml

Interviews

Semi-structured interviews were conducted with patients and staff. The development of the interview guide was iterative; as data collection continued, the content of the guide evolved in order to explore emerging themes. In particular, the interview guide evolved to explore concepts of uncertainty (and how it is communicated), conversations around and attitudes towards palliative care, trust (between members of the MDT and between doctor and patient) and risk communication (see supplemental file). Informal interviews with staff members of the MDT also took place and were incorporated into written field notes. Pseudonyms are used for reporting data throughout to protect the anonymity of respondents.

Analysis

The data were analysed by one researcher (DWH) and emerging analyses were discussed with CE and BH, following principles of constructivist grounded theory [24]. Only one coder was used because of the complexity of the multiple data sources during this ethnographic study. However emerging concepts and themes were discussed formally in the wider research team. All data sources (MDT meeting, clinic, informal and formal interviews) were analysed using the same coding framework. The codes used were conceptual, rather than descriptive, and labels were derived completely from the data, not pre-determined. Line by line coding produced an initial coding framework: the emerging analysis was used during axial coding to guide further sampling and further development of the coding framework. Hence coding was both inductive and deductive and when the coding framework was altered, all transcripts were re-coded. The coding was organised using the NVivo computer package. Emerging findings (and 'memos') were formally discussed in the research team to develop the data analysis and guide subsequent analysis and data sampling

For peer review only - http://bmjopen.bmj.com/site/about/guidelines.xhtml

Results

The research was conducted in three head and neck cancer (HNC) centres in the north east of England. A total of 35 MDT meetings and 37 clinic appointments MDT meetings and clinics were observed for 30 patients (23 males and seven females, aged 38-87 years). Additionally 23 interviews were conducted with patients and nine interviews with MDT members (see table one). In all centres, the MDT meeting took place without the patient present and was attended by surgeons, oncologists, radiologists, pathologists, speech and language therapists, dieticians and administrative staff. Following the meeting, one surgeon met with the patient in clinic. Sometimes other members were present with the surgeon, and other times they were alone. If considering non-surgical options, the patient would meet an oncologist. Each MDT would discuss between 10 and 30 patients; the majority of these patients were then seen in the accompanying clinic

For peer review only

PATIENTS: Group 1	Centre	Age	Tumour site	Observation		Int 1	Int 2
				MDT	Clinic		
James Cain	A	68	Pharynx	1	1	1	1
Frances Cotton	A	82	Pharynx	1	1	x	x
Philip Vase	A	61	Parotid	1	1	x	x
Fred Barnes	A	71	Lip	1	1	x	x
Deborah Dolphin	A	54	Pharynx	1	1	1	x
Vincent Lowry	A	80	Pharynx	1	1	x	x
David Forcett	A	72	Pinna	1	1	x	x
Stanley Wright	A	87	Pharynx	1	1	1	x
Daniel Carding	A	64	Larynx	1	1	1	x
John Winton	A	61	Larynx	1	1	1	x
Bobby Older	A	52	Pharynx	1	1	x	x
Samuel Black	A	55	Pharynx	1	1	1	1
Keith Down	A	62	Larynx	1	1	1	x
William Runman	B	73	Pharynx	3	1	1	x
Andrew Driver	B	49	Pharynx	1	1	1	x
Donna Childs	B	52	Pharynx	1	1	1	x
David Jobling	B	63	Larynx	1	1	x	x
Sophie Leicester	B	49	Larynx	1	1	x	x
Edward Doman	B	73	Mouth	3	1	1	x
Eric Francais	B	65	Larynx	1	1	1	x
Gary Duck	B	57	Pharynx	1	1	x	x
Jean Dixon	B	63	Pharynx	1	2	1	1
Jane Doe	C	69	Pharynx	1	2	1	x
Margaret Brigstock	C	81	Mandible	1	2	x	x
Roy Dayson	C	60	Pharynx	1	1	1	x
Dana O'Malley	C	67	Pharynx	1	1	x	x
Gary Nicholson	C	46	Pharynx	1	2	x	x
Tracey Burnham	C	38	Larynx	1	1	x	x
James Matfield	C	70	Larynx	1	4	1	x
David Dale	C	84	Larynx	1	1	x	x
**PATIENTS: Group 2 (interview only)**							
Kevin Hair	A	82	Pharynx				
David Newman	A	57	Larynx				
Frank Sunnyman	A	52	Pharynx				
Phil Gardener	B	65	Larynx				
**STAFF (interview only)**		**Staff role**					
Mr Red	A	ENT surgeon					
Dr Orange	A	Oncologist					
Mr Surton	A	Maxillofacial surgeon					
Miss Salt	A	Speech and Language Therapist					
Tessa Darling	A	Clinical Nurse Specialist					
Mr Halifax	B	Maxillofacial surgeon					
Mr Blaydon	B	ENT surgeon					
Mr North	B	ENT surgeon					
Dr Goodier	C	Oncologist					

For peer review only

For peer review only - http://bmjopen.bmj.com/site/about/guidelines.xhtml

Table one: details of included participants

”Best” treatment

The MDT meeting discussion often tends towards debate on which treatment is “best” for a patient amongst the available options. In the following interview extract, a maxillofacial surgeon describes his view of the aim of the MDT discussion:

> [The team] need to leave the MDT [meeting] with the treatment options ….prioritised. So a rank order of [the] best treatment clinically – slightly irrespective of the patient’s wishes. From a clinical point of view to try and get best outcome, this would be our first, this would be our second, this would be third and fourth and fifth. Then you discuss it with the patient and say, “This is what we think.”

In this data extract, the surgeon clearly states his view that the aim of the MDT discussion is to decide the “clinically” best treatment for the patient and even goes as far to say that this could be “irrespective” of the patient’s wishes. Teams frequently conclude their discussion of treatment options in the MDT meeting with an agreement for the recommendation (ie the MDT’s perception of “best” treatment). This recommendation is to be delivered to the patient. In the following extract, the MDT members are discussing the merits of surgery (laser) vs radiotherapy

> Mr Black (ENT surgeon): I have a database of the [laser resections] I have done …. tonsil and soft palate tumours, and it’s just….it’s something we need to take notice of
>
> Mr Red (ENT surgeon): Yeah, I think we’ll need to, we’ll have to discuss it another time or we’ll take up the whole morning on one case. But, I think there are arguments for and against...
>
> Dr Orange (oncologist): I would suggest he has radiotherapy, because he will have a slightly better functional outcome, and he’s 80 and …because of his age, and because of the possibly better function….would you Dr Yellow?
>
> Dr Yellow (oncologist): Yes

For peer review only - http://bmjopen.bmj.com/site/about/guidelines.xhtml

> Mr Red: I think there is a consensus view of the MDT, would be for radiotherapy
>
> MR Black: OK
>
> (Observation, MDT meeting)

Although, during this discussion, options of radiotherapy and laser were available to the patient, the position of the MDT meeting was to provide a recommendation for radiotherapy. Here, we see the members of the MDT preparing their 'party line' which is to be delivered to the patient in clinic. This recommendation for "best treatment" is often conveyed to the patient on its own or in preference to other options

The "cycle of paternalism"

Anxious patients, faced with complex decisions can lead leads to patients endorsing the paternalistic approach as they are given little or no information about the available treatment choices and therefore tend to delegate responsibility of the decision to the clinician:

> Pt: You know, they're the doctors, they're the professional people. And I'm just Joe Bloggs off the street. ...For a lot of years, I was a steel erector. I wouldn't expect you as a doctor coming along and doing what I could do. Do I?
>
> Interviewer: You feel a decision should be the doctor's decision?
>
> Pt: Oh, definitely, without a doubt. It's got to be the doctor's decision. How could I make a decision like that?

Here, the patient delegates the decision to the MDT without question; he is allowing the MDT's assessment of 'best' treatment to act as the sole basis for a treatment decision. If decision delegation is accepted as the method by which MDTs convey and make decisions, a paternalistic decision making process results. In this model, the patient accepts that the MDT's assessment of 'best' (and hence the treatment recommendation) is appropriate. It creates a "cycle of paternalism" with grateful patients accepting firm recommendations from clinicians and clinicians reassured that they are doing their best for their patients.

For peer review only - http://bmjopen.bmj.com/site/about/guidelines.xhtml

Delivery of the MDT treatment recommendation

Firm MDT recommendations can sometimes place the clinician in a difficult position when discussing options. The following extract is the clinic appointment for Vincent Lowry (the MDT meeting extract was included above). Here Mr Black (who favoured laser in the MDT meeting, referred to here as "surgery") was delivering the MDT recommendation for radiotherapy to the patient:

> Mr Black: After a lot of discussion, the consensus…. would be to give you radiation therapy…. that was what we jointly decided. And we think with that treatment there is a very good chance of controlling your disease completely….
>
> Pt: Well, I'll do as you say
>
> Daughter: So there's no other operation, it would just be radiotherapy?
>
> Mr Black: We discussed this at length at the meeting…. and the majority of people… felt that to be frank, except for me, felt that radiation would be the way forward. And…. that's what we are offering to you as first line treatment. Unless you have any reservations, then we can think about other options.
>
> Pt: I'll do as you say….
>
> Daughter: Right. So he would have to come into hospital every day? He's a really bad traveller…
>
> Pt: You know when I come here I get all tensed up and travelling….
>
> Mr Black: Really? Is it likely you may then stop the treatment midway for whatever reason, because that would backfire very badly.
>
> Pt: I wouldn't do that
>
> Mr Black: I know you asked about the surgical option. I promised people I wouldn't say anything. But it is feasible to take it out surgically, and there is an option available, but the consensus at the MDT was to go ahead with radiation. Unless, as a family or yourself very strongly object to it and feel that you can't go ahead with that, then of course the surgical option is always there. But as a group we felt that the best way forward was to offer you radiation
>
> Pt: Well. I'll go with you
>
> (Observation, MDT Clinic)

For peer review only

The final treatment decision was to deliver radiation, but the interaction above reveals the challenges of being tasked by the MDT to give a single recommendation when it is used in a decision discussion with a patient. Once new information was gleaned from the patient in clinic (being a "really bad traveller") Mr Black struggled with how to deal with the recommendations: was it a rule to be followed? Here, the rigidity of the treatment recommendation acted as a barrier to an open discussion about the treatment options available to the patient and thus inhibited shared decision making.

Patient engagement with MDT recommendations

Modern clinical practice cannot assume that the sole role of the patient is the acceptance of a single firm treatment recommendations. John Winton was a 61 year old patient with an advanced cancer of his larynx. In the MDT it was decided that surgery (total laryngectomy) should be delivered as a single recommendation. Radiotherapy is available, carries a lower chance of cure, but allows him to retain his voice box. The following data are from his clinic appointment:

> Mr Black (ENT surgeon): This tumour in your throat is a fairly big tumour, and it's spread to the neck as well. We believe that there are two possible ways that we can manage this. At some parts of the scan, there is evidence that the tumour may have gone into the Adam's apple cartilage…. If that is the case, surgery would be the only option to get rid of the tumour. But surgery would involve you losing your voicebox, losing part of the swallowing passage, you would need a big neck operation….Once we do the surgery, your speech will be different, you won't be speaking the same. You will have a hole in the centre of your neck, a tracheostomy
>
> Pt: Nah, nah [shakes head]
>
> Mr Black: You wouldn't fancy that?
>
> Pt: No
>
> Mr Black: That's the surgical option. On the other side is the option of radiation therapy
>
> Pt: I would rather take a chance with that

For peer review only - http://bmjopen.bmj.com/site/about/guidelines.xhtml

(Observation, MDT Clinic)

Here, and throughout the course of this consultation, the patient made a decision to reject surgery, which reduces his length of survival from his cancer in order to preserve his voice box. He was adamant he did not want a complete removal of the voice box and part of the throat (pharygolaryngectomy) and the decision was eventually made to use radiotherapy. However, in the subsequent interview, Mr Winton discussed the rationale for his decision:

> Pt: Well you see my mother died of cancer… my father died of cancer, and I've seen the way cancer works. I'm not being cheeky…. once they cut you open, it's like your letting fresh air into a bulb, it then just spreads, and they stitch you back up again and "We've cured it", right, for how long? And then it comes back again…
>
> Interviewer: And what's important to you when you're making that decision?
>
> Pt: Surviving as long as I can…, I mean if you get the year, 18 months it's better than getting two weeks isn't it?

His consultation, which was limited in exploring what mattered to him, leads to a decision that is potentially at odds with his aspirations revealed above. His aim of treatment (survival) is not matched by the actual treatment decision (radiotherapy). This patient perspective could not be incorporated into the previous MDT discussion (which happened before the clinic appointment), but equally the subsequent clinic appointment did not explore his preferences and what underpinned them, risking a treatment decision at odds with his preferences and values. If Mr Black had explored the options for treatment with the patient more, this mismatch of treatment preferences and values could have been identified, and perhaps deconstructed. Such information about values and preferences is essential to good shared decision making, however very difficult to incorporate into the MDT decision making structure.

For peer review only - http://bmjopen.bmj.com/site/about/guidelines.xhtml

Discussion

This study has found that patient engagement with the outcome of an MDT discussion (a recommendation for "best" treatment) is problematic.Often patients accept this recommendation in the clinic (perhaps precisely because it is presented as the "best" treatment). However, this acquiescence may be due to the disempowered position in which patients find themselves as they confront a terrifying diagnosis and a myriad of complex decision options. In turn, clinicians often view the acceptance of an MDT recommendation as delegation of the decision by the patient to the clinician, an assumption which can promulgate a 'cycle of paternalism', where anxious patients have little real choice other than to accept the clear guidance offered by the expert team. However, limiting patient involvement to acceptance or rejection of a firm recommendation leads to decisions which are not in line with patient values and can not be considered patient-centred, shared decision making.

The rigidity of the MDT recommendation can act as a barrier to an open discussion of the available options. If the patient role is limited to either acceptance or refusal of a single recommendation, true engagement is impossible. A truncated discussion of a single MDT recommendation for treatment prohibits shared decision-making using the "three talk model" , as central to this model is a discussion of the options for treatment. A shared decision-making consultation allows the patient and clinician to explore the risks, benefits and consequences of a treatment alternatives; a move from initial to informed preferences; and exploration of patient values to reach a shared decision [27].

The structure of MDT working has not significantly changed since its inception in 1996. NHS patients rarely attend their MDT meetings, modern cancer care mandates that all

For peer review only - http://bmjopen.bmj.com/site/about/guidelines.xhtml

patients are discussed in this setting[28] and interventions to increase the number of patients discussed in an MDT are still sought after[29].

The MDT recommendation

If the MDT meeting and clinic follow a paternalistic pathway, the way in which their recommendation is used is clear: it is delivered to the patient with an assumption that it will be accepted. In the paternalistic tradition, physicians are considered to be best placed to evaluate the trade-offs and pitfalls of treatment, and applied these to the decision process based on their evaluation of the best interests of the patient [30]. However, often in cancer care (particularly head and neck cancer), treatment options are available for a patient: which of these is "best" depends on the value you apply to the various aspects of the treatment. For example, is the priority of treatment cure or preservation of quality of life? What functional impact will a patient endure to achieve tumour control? What aspects of functional decline (such as speech, swallow or aesthetics) are most important? The answers to these questions are based on values: clinicians and patient do not share values [31-33]. Thus MDTs must ensure that treatment decisions are driven by patient values. Although patients may justifiably actively delegate some or all of the responsibility for the decision to the MDT members, at the same time, the MDT have a duty to ensure that this is not due to disempowerment or lack of access to the information required to take an active part in decision making. Hence the clinician has a role to, at the very least, support the patient to understand what is important to them before accepting the role as decision maker on the patient's behalf

Outwith the MDT decision process, a treatment recommendation from an individual clinician can be modified depending on the ongoing interaction with the patient and the preferences expressed. An MDT recommendation, on the other hand, is problematic for MDT members who attempt to combine it with the values or preferences of the patient. Is it set in stone, an

For peer review only - http://bmjopen.bmj.com/site/about/guidelines.xhtml

obligatory 'best' which must be adhered to? If the patient disagrees with the recommendation, what action should the MDT member take? In this way, MDT recommendations are inflexible, especially in the light of new information from the patient which was not clear or known in the MDT meeting. In other words, information about values and preferences are vital to a shared decision but difficult to incorporate into the MDT decision making structure. As we have previously described [21], MDTs often build the "evidential patient" in the MDT meeting discussion. This may include information about a patient's values and preferences, but these are impossible to incorporate into a meeting discussion without the patient present and without making assumptions about the patient.

Modernising MDT decision-making

If we are to modernise the MDT decision making structure to improve patient involvement, the role of the MDT discussion and the structure of the clinic must recognise that patients often "distribute" decisions. Rapley [34] describes how patients demonstrate a 'relational autonomy' by distributing their decision amongst people, encounters, places and information sources. Promoting relational autonomy means that involving patients in decisions requires more than presenting options and awaiting a verdict, instead emphasising the importance of the interaction with the clinician, encouraging questions, correcting misunderstanding, constructing preferences and allowing disagreement [35]. Indeed, the MDT decision-making structure gives ample opportunity for MDT members to distribute their decision amongst colleagues, but does not afford the same opportunity to patients

If the patient is to be a true participant in shared decision making, an alternative model of MDT decision making is required. Some teams have explored the idea of a patient attending their own MDT meeting, with many patients reporting a positive experience[36]: this idea is popular amongst patient advocates [37], but clinicians have mixed views [10 37 38]. Small studies

For peer review only - http://bmjopen.bmj.com/site/about/guidelines.xhtml

have concluded that patients attending their own MDT allows for better information giving [39] [40]and the opportunity to ask questions and contribute information such as preference [41]; however included patients may have higher health literacy[42] raising the possibility that including patients has potential to widen health inequality. MDT members often feel that patients attending their own meeting would inhibit the discussion and cause patient anxiety [37]; relationships within the MDT are often longstanding with pre existing hierarchies which can present barriers to new user integration[43]Nevertheless, if patients are to be included in MDT meetings, clarity is required on how patients, their supporters and healthcare teams are supported to make it a positive and worthwhile experience[44].

Of key importance is that the MDT meeting is not a discussion of which option is 'best' for a particular patient, but should instead aim to determine which valid treatment options are available. In particular, palliative options (or options of 'doing nothing') are often inadequately explored [45]. Clinic structures should be flexible to allow patients to distribute their decision-making amongst information sources and people. The patient may be enabled to come to the initial consultation more informed and prepared for the discussion. There may be a role for pre MDT clinic with the patient meeting a surgeon, oncologist or specialist nurse, or a post MDT clinic to convey options and explore values and preferences, maybe with more than one clinician. The MDT meeting may take place in a small 'combined clinic' setting around the interaction with the patient. The MDT members provide support, resources and personnel to discuss the treatment options, communicate the risk and uncertainty, elicit values and explore them; a decision aid may support this work [46]. The team may consider providing an individual who is independent of the clinical team to act as a decision coach or navigator [47]. MDT members should be encouraged to update their training in supporting patients in shared decision making, consent and communication.This study provides a novel and rich account of the difficulties that patients face when making a decision in the context of

For peer review only - http://bmjopen.bmj.com/site/about/guidelines.xhtml

an MDT. MDT decision making is mandated internationally however the specific structure of the decision process varies widely. Although the structure presented here (MDT meeting without a patient present, recommendation delivered to the patient separately) is common, other models of MDT decision making may not face similar challenges. Also, ethnographic methods, in providing depth to explore a smaller number of concepts in more detail, may lack the breadth of findings to make this piece of work widely applicable. Nevertheless, whilst the setting may not be universally generalisable, we hope that the emergent conclusions will be.

It is time for the development and design of alternative models of team decision making which have a central role for the patient. Further work to develop new model of delivering team decision-making would be multifactorial, incorporating the development of the structure of the MDT meeting and clinic, support and training for MDT members and patients and the development of tools to be used in combination with team decisions. Qualitative approaches should explore stakeholders' views of intervention components, which should be co-designed with patients. Evaluation of such interventions requires novel trial design, comparing methods of decision making and evaluating decision quality. MDT decision making is now ubiquitous and therefore the urgent need of reform to meet the principles of shared decision making should be a priority for clinical teams and cancer researchers.

Author Contributions

DW Hamilton
Protocol development, ethical approval, data gathering, data analysis, manuscript preparation

B Heaven
Protocol development, ethical approval, data analysis, manuscript preparation

R G Thomson
Protocol development, data analysis, manuscript preparation

JA Wilson

For peer review only - http://bmjopen.bmj.com/site/about/guidelines.xhtml

Protocol development, data analysis, manuscript preparation

C Exley
Protocol development, ethical approval, data analysis, manuscript preparation

Competing interests

All authors have completed the Unified Competing Interest form at www.icmje.org/coi_disclosure.pdf (available on request from the corresponding author) and declare that DWH, BH, RT, JAW and CE have no relationships with any companies that might have an interest in the submitted work in the previous 3 years; and DWH, BH, RT, JAW and CE have no non-financial interests that may be relevant to the submitted work

Funding

This study represents independent research funded by the National Institute of Health Research. The views expressed are those of the author(s) and not necessarily those of the National Health Service, the National Institute of Health Research or the Department of Health

Data sharing statement

No additional data available

References

1. Licitra L, Keilholz U, Tahara M, et al. Evaluation of the benefit and use of multidisciplinary teams in the treatment of head and neck cancer. *Oral Oncol* 2016;59:73-9. doi: 10.1016/j.oraloncology.2016.06.002

2. Calman KC, Hine D. A Policy Framework for Commissioning Cancer Services In: Health Do, ed., 1995.

3. Kelly SL, Jackson JE, Hickey BE, et al. Multidisciplinary clinic care improves adherence to best practice in head and neck cancer. *American journal of otolaryngology* 2013;34(1):57-60. doi: 10.1016/j.amjoto.2012.08.010

4. Patil RD, Meinzen-Derr JK, Hendricks BL, et al. Improving access and timeliness of care for veterans with head and neck squamous cell carcinoma: A multidisciplinary team's approach. *Laryngoscope* 2016;126(3):627-31. doi: 10.1002/lary.25528

5. Starmer H, Sanguineti G, Marur S, et al. Multidisciplinary head and neck cancer clinic and adherence with speech pathology. *Laryngoscope* 2011;121(10):2131-5. doi: 10.1002/lary.21746

6. Taylor C, Munro AJ, Glynne-Jones R, et al. Multidisciplinary team working in cancer: what is the evidence? *Bmj* 2010;340:c951. doi: 10.1136/bmj.c951

For peer review only - http://bmjopen.bmj.com/site/about/guidelines.xhtml

For peer review only

7. Wang YH, Kung PT, Tsai WC, et al. Effects of multidisciplinary care on the survival of patients with oral cavity cancer in Taiwan. *Oral Oncol* 2012;48(9):803-10. doi: 10.1016/j.oraloncology.2012.03.023

8. Birchall M, Bailey D, King P, et al. Effect of process standards on survival of patients with head and neck cancer in the south and west of England. *British journal of cancer* 2004;91(8):1477-81. doi: 10.1038/sj.bjc.6602118

9. Friedland PL, Bozic B, Dewar J, et al. Impact of multidisciplinary team management in head and neck cancer patients. *British journal of cancer* 2011;104(8):1246-8. doi: 10.1038/bjc.2011.92

10. NCAT. Multidisciplinary team members' views about MDT working: results from a survey commissioned by the National Cancer Action Team. London Kings College, 2009.

11. Rajan S, Foreman J, Wallis MG, et al. Multidisciplinary decisions in breast cancer: does the patient receive what the team has recommended? *British journal of cancer* 2013;108(12):2442-7. doi: 10.1038/bjc.2013.267

12. Blazeby JM, Wilson L, Metcalfe C, et al. Analysis of clinical decision-making in multi-disciplinary cancer teams. *Annals of oncology : official journal of the European Society for Medical Oncology / ESMO* 2006;17(3):457-60. doi: 10.1093/annonc/mdj102

13. Wood JJ, Metcalfe C, Paes A, et al. An evaluation of treatment decisions at a colorectal cancer multi-disciplinary team. *Colorectal disease : the official journal of the Association of Coloproctology of Great Britain and Ireland* 2008;10(8):769-72. doi: 10.1111/j.1463-1318.2007.01464.x

14. Taylor C, Atkins L, Richardson A, et al. Measuring the quality of MDT working: an observational approach. *BMC cancer* 2012;12:202. doi: 10.1186/1471-2407-12-202

15. Rowlands S, Callen J. A qualitative analysis of communication between members of a hospital-based multidisciplinary lung cancer team. *Eur J Cancer Care* 2013;22(1):20-31. doi: 10.1111/ecc.12004

16. Lanceley A, Savage J, Menon U, et al. Influences on multidisciplinary team decision-making. *Int J Gynecol Cancer* 2008;18(2):215-22. doi: DOI 10.1111/j.1525-1438.2007.00991.x

17. Soukup T, Murtagh G, Lamb BW, et al. Degrees of Multidisciplinarity Underpinning Care Planning for Patients with Cancer in Weekly Multidisciplinary Team Meetings: Conversation Analysis. *J Multidiscip Healthc* 2021;14:411-24. doi: 10.2147/JMDH.S270394 [published Online First: 2021/02/27]

18. Kidger J, Murdoch J, Donovan JL, et al. Clinical decision-making in a multidisciplinary gynaecological cancer team: a qualitative study. *Bjog-Int J Obstet Gy* 2009;116(4):511-17. doi: DOI 10.1111/j.1471-0528.2008.02066.x

19. Horlait M, Baes S, Dhaene S, et al. How multidisciplinary are multidisciplinary team meetings in cancer care? An observational study in oncology departments in Flanders,

For peer review only - http://bmjopen.bmj.com/site/about/guidelines.xhtml

Belgium. *J Multidiscip Healthc* 2019;12:159-67. doi: 10.2147/JMDH.S196660 [published Online First: 2019/03/14]

20. Horlait M, De Regge M, Baes S, et al. Exploring non-physician care professionals' roles in cancer multidisciplinary team meetings: A qualitative study. *PLoS One* 2022;17(2):e0263611. doi: 10.1371/journal.pone.0263611 [published Online First: 2022/02/04]

21. Hamilton DW, Heaven B, Thomson RG, et al. Multidisciplinary team decision-making in cancer and the absent patient: a qualitative study. *BMJ Open* 2016;6(7):e012559. doi: 10.1136/bmjopen-2016-012559 [published Online First: 2016/07/23]

22. Taylor C, Finnegan-John J, Green JS. "No decision about me without me" in the context of cancer multidisciplinary team meetings: a qualitative interview study. *BMC health services research* 2014;14:488. doi: 10.1186/s12913-014-0488-2

23. Strauss AL. Continual permutations of action. New York: Aldine de Gruyter 1993.

24. Charmaz K, editor. *Constructing Grounded Theory. A Practical Guide Through Qualitative Analysis*. London: SAGE, 2006.

25. Strauss AL, Corbin JM. Basics of qualitative research : grounded theory procedures and techniques. Newbury Park, Calif.: Sage Publications 1990.

26. Dey I. Grounding grounded theory : guidelines for qualitative inquiry. San Diego: Academic Press 1999.

27. Elwyn G, Durand MA, Song J, et al. A three-talk model for shared decision making: multistage consultation process. *Bmj* 2017;359:j4891. doi: 10.1136/bmj.j4891 [published Online First: 2017/11/08]

28. NICE. Improving Outcomes in Head and Neck Cancer - the Manual. London, 2004.

29. Maharaj AD, Evans SM, Zalcberg JR, et al. Barriers and enablers to the implementation of multidisciplinary team meetings: a qualitative study using the theoretical domains framework. *BMJ quality & safety* 2021;30(10):792-803. doi: 10.1136/bmjqs-2020-011793 [published Online First: 2020/11/29]

30. Charles C, Gafni A, Whelan T. Decision-making in the physician-patient encounter: revisiting the shared treatment decision-making model. *Social science & medicine* 1999;49(5):651-61. doi: S0277953699001458 [pii] [published Online First: 1999/08/19]

31. Hamilton DW, Bins JE, McMeekin P, et al. Quantity vs quality of life in laryngeal cancer: a time trade off study. *Clinical Otolarygology* 2012;37(Suppl. 1):108.

32. Hamilton DW, Pedersen A, Blanchford H, et al. A comparison of attitudes to laryngeal cancer treatment outcomes: A time trade-off study. *Clinical Otolaryngology* 2018;43(1):117-23. doi: 10.1111/coa.12906

For peer review only - http://bmjopen.bmj.com/site/about/guidelines.xhtml

33. Slevin ML, Stubbs L, Plant HJ, et al. Attitudes to chemotherapy: comparing views of patients with cancer with those of doctors, nurses, and general public. *Bmj* 1990;300(6737):1458-60. [published Online First: 1990/06/02]

34. Rapley T. Distributed decision making: the anatomy of decisions-in-action. *Sociol Health Illn* 2008;30(3):429-44. doi: SHIL1064 [pii]

10.1111/j.1467-9566.2007.01064.x [published Online First: 2008/01/16]

35. Entwistle VA, Carter SM, Cribb A, et al. Supporting patient autonomy: the importance of clinician-patient relationships. *J Gen Intern Med* 2010;25(7):741-5. doi: 10.1007/s11606-010-1292-2

36. Diekmann A, Heuser C, Ernstmann N, et al. How do breast cancer patients experience multidisciplinary tumor conferences? - A description from the patient perspective. *Breast* 2019;44:66-72. doi: 10.1016/j.breast.2018.12.012 [published Online First: 2019/01/23]

37. Butow P, Harrison JD, Choy ET, et al. Health professional and consumer views on involving breast cancer patients in the multidisciplinary discussion of their disease and treatment plan. *Cancer* 2007;110(9):1937-44. doi: 10.1002/cncr.23007

38. Heuser C, Diekmann A, Schellenberger B, et al. Patient Participation in Multidisciplinary Tumor Conferences from the Providers' Perspective: Is It Feasible in Routine Cancer Care? *J Multidiscip Healthc* 2020;13:1729-39. doi: 10.2147/JMDH.S283166 [published Online First: 2020/12/05]

39. Bellardita L, Donegani S, Spatuzzi AL, et al. Multidisciplinary Versus One-on-One Setting: A Qualitative Study of Clinicians' Perceptions of Their Relationship With Patients With Prostate Cancer. *Journal of oncology practice / American Society of Clinical Oncology* 2011;7(1):e1-5. doi: 10.1200/JOP.2010.000020

40. Choy ET, Chiu A, Butow P, et al. A pilot study to evaluate the impact of involving breast cancer patients in the multidisciplinary discussion of their disease and treatment plan. *Breast* 2007;16(2):178-89. doi: 10.1016/j.breast.2006.10.002

41. Bohmeier B, Schellenberger B, Diekmann A, et al. Opportunities and limitations of shared decision making in multidisciplinary tumor conferences with patient participation - A qualitative interview study with providers. *Patient education and counseling* 2021;104(4):792-99. doi: 10.1016/j.pec.2020.09.007 [published Online First: 2020/10/15]

42. Heuser C, Diekmann A, Kowalski C, et al. Health literacy and patient participation in multidisciplinary tumor conferences in breast cancer care: a multilevel modeling approach. *BMC cancer* 2019;19(1):330. doi: 10.1186/s12885-019-5546-z [published Online First: 2019/04/10]

43. Martin GP, Finn R. Patients as team members: opportunities, challenges and paradoxes of including patients in multi-professional healthcare teams. *Sociol Health Illn* 2011;33(7):1050-65. doi: 10.1111/j.1467-9566.2011.01356.x

For peer review only - http://bmjopen.bmj.com/site/about/guidelines.xhtml

44. Schellenberger B, Heuser C, Diekmann A, et al. Questions and emotional expressions from patients and companions while participating in multidisciplinary tumor conferences in breast and gynecological cancer centers. *Patient education and counseling* 2021 doi: 10.1016/j.pec.2021.12.010 [published Online First: 2021/12/27]

45. Nelson A, Longo M, Byrne A, et al. Chemotherapy decision-making in advanced lung cancer: a prospective qualitative study. *BMJ Support Palliat Care* 2020 doi: 10.1136/bmjspcare-2020-002395 [published Online First: 2020/08/23]

46. Stacey D, Legare F, Lewis K, et al. Decision aids for people facing health treatment or screening decisions. *Cochrane Db Syst Rev* 2017(4) doi: ARTN CD001431

10.1002/14651858.CD001431.pub5

47. Stacey D, Murray MA, Legare F, et al. Decision coaching to support shared decision making: a framework, evidence, and implications for nursing practice, education, and policy. *Worldviews Evid Based Nurs* 2008;5(1):25-35. doi: WVN108 [pii]

10.1111/j.1741-6787.2007.00108.x [published Online First: 2008/02/13]

For peer review only

Staff Interview Schedule

Introduce researcher and purpose of the study

Obtain consent to proceed and to record the conversation

- What do you see as the best way of making decisions in head and neck cancer?
- The MDT
    - What is it for? What is its primary aim?
    - What works well, and what works not so well?
    - How could it be improved?
    - No decision about me without me in the MDT…. Is this possible?
- Treatment decision
    - Why do you regard a treatment as the best for a patient?
    - What factors do you take into account when making treatment decisions?
- Patient involvement in decisions/shared decision making
    - What does it mean to you?
    - Do you think there are barriers to patient involvement? What are they? Why do they exist?
    - Why do clinicians struggle to involve their patients effectively?
    - Do you think we need to improve patient involvement? Why?
- Uncertainty/conflict
    - Is it a problem or is it healthy/required?
    - How should uncertainty and conflict be presented to the patient? Should they know that you don't know?
    - If there are options, how should they be communicated?
- Decision for treatment
    - What is the role of the patient in the treatment decision?
    - How much of a role should the patient have? Can patients know enough?
    - How much of a role should a patient have in the decision
        - Should we give power of vito?
        - Should you allow a patient to make a decision which is considered wrong?
        - how much should a patient know before treatment
    - How much of a role should the family have
- Palliation/prognosis
    - Do we palliate enough? Do we treat too many people radically?
    - What are the barriers to good palliation?
    - Do you think we have a range of palliative options available to us?
    - Do you have prognostic information available to you?
    - Would you use this information if it was available? How would you use it?

For peer review only

New patient interview 1 schedule

Introduce researcher and purpose of the study

Obtain consent to proceed and to record the conversation

The interview will cover the following broad areas:

- Previous experience of making healthcare decisions
  - What does shared decision making mean?
  - Have you had to make big healthcare decisions before?
- Experience of making this decision
  - What was your process of making the decision? Talk me though it
  - What did you draw upon?
  - What factors did you take into account?
  - Did you turn to anybody/anything for information or help?
  - How did you come to your final decision?
  - What was the MDT like?
  - Did you understand what was going on?
  - Could it be improved?
- Information given about the disease and treatment options
  - Do you think you had enough information?
  - Where do you get your information from?
  - Do you think you need more information?
  - Did you understand all the information
- Patient involvement in the decision about care
  - Should patients be involved in decisions about their care?
  - How should we involve patients?
  - Were you involved? How?
  - Would you have liked more say/less say?
  - How this made the participant feel, and further exploration
- What are your expectations of treatment?
  - Are you expecting side effects? What sort?
  - Do you have any idea of your prognosis? Do you want to know?
  - Where will you be in 6 months time?
- In the perfect world, how do you think decisions should be made?

For peer review only

New patient interview 2 schedule/retrospective patient interview schedule

Introduce researcher and purpose of the study

Obtain consent to proceed and to record the conversation

- Experience of the treatment
  - What has happened so far, story of the treatment
  - Correlation with expectations – worse, better
- New normal
  - Do you have a good quality of life?
  - How do you cope with swallowing?
  - How is your voice, how do you get on with communication?
- MDT and decision process
  - When you look back did you understand the treatment decision?
  - Did you understand the consequences of the decision?
  - Did you have enough information to base the decision on?
  - Should they have allowed you more or less control over the decision?
  - Do you wish the decision had been made differently?
  - Do you feel you made the right or the wrong decision?
  - Do you have any regrets?
  - How could they improve the clinic/the decision process?
  - In the perfect world, how do you think treatment decisions should be made?
- Palliation/prognosis
  - Were you ever aware of the chances of the treatment being successful?
  - Would you want to be aware?
  - Was palliation ever an option for you? Is it now?
- Information giving
  - Do you think you had enough information about treatment?
  - Did you understand what you were entering yourself into?
  - Should they give more information? Could they?

For peer review only

Topic	Page	Short description
1: Title	1	How do patients make decisions in the context of a multidisciplinary team: an ethnographic study of four head and neck cancer centres
2: Abstract	2-3	Included in manuscript
3: Problem formulation	4-5	MDT working is common and expensive, but the impact on shared decision making and patient involvement in decisions remains relatively unexplored
4: Purpose or research question	4-5	To examine critically patient engagement in the MDT treatment decision making process in head and neck cancer, and to evaluate the experience and practice of decision-making by patients and clinicians
5: Qualitative approach and research paradigm	5-7	Ethnographic methods (non-participant observation and semi-structured interviews). Analysis followed principles of constructivist grounded theory
6 Researcher characteristics and reflexivity	5	All data were collected by the lead author (DWH) whilst performing his PhD. At the time, he was a trainee surgeon and had taken time out of programme for research. The dual role of surgical trainee and researcher allowed easy access to the setting and language, making ethnography and observations easier and potentially more 'natural'. Co-authors BH and CE were involved in analysis in order to minimise the effect of the researcher's status on the emerging conclusions
7 Context	6&7	The research was conducted in four head and neck cancer centres in the north east of England. In all centres, the MDT meeting took place without the patient present; following this, one or more members of the MDT met with the patient in clinic
8 Sampling strategy	7	Initial sampling aimed to recruit patients who had a treatment decision to make about their care, or where more than one treatment option was available to the patient. . Purposive sampling guided the sampling strategy to explore emerging concepts with data collection and analysis occurring in tandem. Thus, further sampling was guided by the emerging analysis and continued until a state of theoretical sufficiency was achieved.
9 Ethical issues pertaining to human subjects	5	Ethical approval was gained from the NHS Research Ethics Newcastle and North Tyneside 2 committee (reference 11/NE/0200) in September 2011 and all necessary local Research and Development governance permissions were obtained. All participants provided informed consent.
10 Data collection methods	6	Non-participant observation; semi structured interviews with patients and staff; field notes; reflective notes. Iterative process with emerging themes explored through further data collection. All

For peer review only

		emerging analysis and memos discussed with co-authors (CE and BH)
11 Data collection instruments and technologies	6	Interview guides (iteratively developed) used for interviews. Field notes and notes from informal discussions. Audiorecording and word-for-word transcription of all formal interviews, MDT meeting and MDT clinic
12 Units of study	6	34 observations of MDT meetings and 37 clinic appointments (for 30 patients; 13 of these patients were interviewed once, three were interviewed twice). Interviews with four further treated patients and nine members of staff.
13 Data processing	6-7	All audiorecordings were transcribed word for word and anonymised. All reflective notes and field notes were anonymised. Patients and staff given pseudonyms. MDT meeting data transcribed by the main author (DWH) and all recording transcribed by others were checked by DWH. Audio recordings destroyed after transcription. Data kept on University computers, password protected
14 Data analysis	7	The data were analysed by one researcher (DWH) and emerging analyses were discussed with CE and BH, following principles of constructivist grounded theory. All data sources (MDT meeting, clinic, informal and formal interviews) were analysed using the same coding framework. Line by line coding produced an initial coding framework: the emerging analysis was used during axial coding to guide further sampling and further development of the coding framework; when the coding framework was altered, all transcripts were re-coded. The codes used were conceptual, rather than descriptive, and labels were derived completely from the data, not pre-determined. The coding was organised using the NVivo computer package. Emerging findings (and 'memos') were discussed in the research team (particularly with CE and BH) to develop the data analysis and guide subsequent analysis and data sampling
15 Techniques to enhance trustworthiness	7	All emerging themes and analysis were discussed in the research team with CE and BH involved in analysis. Findings presented in departmental meetings
16 Synthesis and interpretation	7-12	The outcome of an MDT discussion is frequently a "in the clinic as they confront a terrifying diagnosis and a myriad of complex decision options. In turn,.
17 Links to empirical data	7-12	Data presented in the results section linked to empirical data throughout
18 Integration with prior work, implications, transferability and contributions to the field	12-16	The discussion section discusses the findings in the context of prior work and the contributions to the field

For peer review only

19 Limitations	3, 18-19	Strengths and limitations are discussed in the discussion section and there is a list of strengths and limitations at the beginning of the manuscript
20 Conflict of interest	16	All authors have completed the Unified Competing Interest form at www.icmje.org/coi_disclosure.pdf (available on request from the corresponding author) and declare that DWH, BH, RT, JAW and CE have no relationships with any companies that might have an interest in the submitted work in the previous 3 years; and DWH, BH, RT, JAW and CE have no non-financial interests that may be relevant to the submitted work
21 Funding	17	This study represents independent research funded by the National Institute of Health Research. The views expressed are those of the author(s) and not necessarily those of the National Health Service, the National Institute of Health Research or the Department of Health

For peer review only

BMJ Open

How do patients make decisions in the context of a multidisciplinary team: an ethnographic study of four head and neck cancer centres in the north of England

Journal:	*BMJ Open*
Manuscript ID	bmjopen-2022-061654.R2
Article Type:	Original research
Date Submitted by the Author:	08-Jul-2022
Complete List of Authors:	Hamilton, David; Newcastle University, Population Health Sciences Institute; Newcastle Upon Tyne Hospitals NHS Foundation Trust, ENT<br>Heaven, Benjamin; Newcastle University, Population Health Sciences Institute<br>Thomson, Richard; Newcastle University, Population Health Sciences Institute<br>Wilson, Janet; Newcastle University, Population Health Sciences Institute<br>Exley, Catherine; Newcastle University, Population Health Sciences Institute
**Primary Subject Heading**:	Patient-centred medicine
Secondary Subject Heading:	Communication, Ear, nose and throat/otolaryngology, Health services research, Qualitative research
Keywords:	Organisation of health services < HEALTH SERVICES ADMINISTRATION & MANAGEMENT, Quality in health care < HEALTH SERVICES ADMINISTRATION & MANAGEMENT, Head & neck tumours < ONCOLOGY, Head & neck surgery < OTOLARYNGOLOGY, QUALITATIVE RESEARCH

SCHOLARONE™
Manuscripts

I, the Submitting Author has the right to grant and does grant on behalf of all authors of the Work (as defined in the below author licence), an exclusive licence and/or a non-exclusive licence for contributions from authors who are: i) UK Crown employees; ii) where BMJ has agreed a CC-BY licence shall apply, and/or iii) in accordance with the terms applicable for US Federal Government officers or employees acting as part of their official duties; on a worldwide, perpetual, irrevocable, royalty-free basis to BMJ Publishing Group Ltd ("BMJ") its licensees and where the relevant Journal is co-owned by BMJ to the co-owners of the Journal, to publish the Work in this journal and any other BMJ products and to exploit all rights, as set out in our licence.

The Submitting Author accepts and understands that any supply made under these terms is made by BMJ to the Submitting Author unless you are acting as an employee on behalf of your employer or a postgraduate student of an affiliated institution which is paying any applicable article publishing charge ("APC") for Open Access articles. Where the Submitting Author wishes to make the Work available on an Open Access basis (and intends to pay the relevant APC), the terms of reuse of such Open Access shall be governed by a Creative Commons licence – details of these licences and which Creative Commons licence will apply to this Work are set out in our licence referred to above.

Other than as permitted in any relevant BMJ Author's Self Archiving Policies, I confirm this Work has not been accepted for publication elsewhere, is not being considered for publication elsewhere and does not duplicate material already published. I confirm all authors consent to publication of this Work and authorise the granting of this licence.

For peer review only

Title: How do patients make decisions in the context of a multidisciplinary team: an ethnographic study of four head and neck cancer centres in the north of England

DW Hamilton
Consultant Otolaryngology, Head and Neck Surgery, Honorary Senior Lecturer
Freeman Hospital, High Heaton, Newcastle upon Tyne NE7 7DN
Population Health Sciences Institute, Newcastle University, Newcastle upon Tyne, NE2 4AX

B Heaven
Formerly Newcastle University, has now left academia

R G Thomson
Emeritus Professor
Population Health Sciences Institute, Newcastle University, Newcastle upon Tyne, NE2 4AX

JA Wilson
Emerita Professor of Otolaryngology, Head and Neck Surgery
Population Health Sciences Institute, Newcastle University, Newcastle upon Tyne, NE2 4AX

C Exley
Professor of Qualitative Health Research
Population Health Sciences Institute, Newcastle University, Newcastle upon Tyne, NE2 4AX

Corresponding Author:
DW Hamilton
Freeman Hospital, High Heaton, Newcastle upon Tyne NE7 7DN
David.hamilton@ncl.ac.uk

Keywords
Decision Making, Shared
Communication
Cancer
Anthropology, Cultural
Informed consent
Autonomy

For peer review only - http://bmjopen.bmj.com/site/about/guidelines.xhtml

Abstract

Objectives

To describe how patients are engaged with cancer decisions in the context of multidisciplinary team (MDT) and how MDT recommendations are operationalised in the context of a shared decision.

Design

Ethnographic qualitative study

Setting

Three head and neck cancer centres in the north of England

Participants

Patients with a diagnosis of new or recurrent head and neck cancer; non-participant observation of 35 MDT meetings and 37 MDT clinics, informal interviews, and formal, semi-structured interviews with 20 patients and 9 MDT staff members

Methods

Ethnographic methods including non-participant observation of MDT meetings and clinic appointments, informal interviews, field notes and formal semi-structured interviews with patients and MDT members

Results

MDT discussions often conclude with a firm recommendation for treatment. When delivered to a patient in clinic this recommendation is often accepted by the patient, but this response may result from the disempowered position in which they find themselves. Whilst patient behaviour may thus appear to endorse clinicians' views that a paternalistic approach is desired by patients (creating a "cycle of paternalism"), the rigidity of the MDT treatment

For peer review only

recommendation can act as a barrier to discussion of options and the exploration of patient values.

Conclusions

The current model of MDT decision making does not support shared decision making and may actively undermine it. A model should be developed whereby the individual patient perspective has more input into MDT discussions, and where decisions are made on potential treatment options rather than providing a single recommendation for discussion with the patient. Deeper consideration should be given to how the MDT incorporates the patient perspective and/or delivers its discussion of options to the patient. In order to achieve these objectives, a new model of MDT working is required.

Word count: 3732

Strengths and limitations of this study

- This ethnographic study provides an in-depth analysis of the complexities of patient involvement and interaction with MDT decision making
- The methods (direct observation and semi structured interviews) allow a rich, data-driven analysis of a complex decision-making environment
- Head and neck cancer involves the trade off of function for survival and is thus a useful model when exploring complex decision making
- As is commonplace in qualitative researcher one researcher led the sampling, collecting and analysis, but the whole team were involved in discussions about interpretation of the data
- Although the structure of MDT decision making discussed here predominates in the UK, the issues faced will not be applicable to all teams

For peer review only - http://bmjopen.bmj.com/site/about/guidelines.xhtml

Introduction

Multidisciplinary team (MDT) decision making is internationally mandated to support appropriate high-quality treatment of patients with cancer [1]. In the UK, MDT working was established following the Calman-Hine report [2] and improves many aspects of cancer treatment such as staging, recruitment to trials [1], adherence to treatment guidelines [3], use of effective evidence-based therapy, timeliness of care [4] and access to the allied members of the healthcare team [5]. However the practice is time consuming and expensive, costing at least £100 million a year in the UK for data preparation and the same amount again for attendance in the UK [6]. To date no MDT cost-benefit analysis has been performed.

MDT members report that consideration of the patient as a person in decision making as a vital part of the decision-making process. In a survey of 2054 MDT members, 95% of respondents felt that "Patient views should always inform the decision-making process" and "Patient views/preferences should be presented to the MDT by somebody who has met the patient" [7]. Omitting patient preference information has an effect on the implementation of MDT recommendations [8-10]. MDT meetings are often dominated by discussion among doctors rather than including other MDT members who may know the patient better or have a more patient-centred perspective [11-14] creating a predominance of the biomedical model of disease [15-17]. This means that the stated aim of many MDT members - to have the patient central to the MDT treatment discussion - is at odds with the reality of the MDT process

We have previously described that if MDT meetings are to become more patient-centred, merely introducing increasing amounts of information about the patient into the MDT is not sufficient [18]. Although we know that the direct viewpoint of the patient within the MDT is lacking [19], there is to date no account of how patients engage with decisions about their treatment in the context of MDTs. This work aims to explore the experience of making

For peer review only - http://bmjopen.bmj.com/site/about/guidelines.xhtml

decisions in the context of an MDT, with a particular emphasis on the patient experience of the decision process.

Methods

This qualitative study used non-participant observation and semi-structured interviews to critically examine how decisions were made in and around the MDT with a particular focus on patient centredness. All data were collected by one researcher (DWH), a head and neck surgeon. Non-participant observation enables the researcher to study participants in their natural environment, and adds value to retrospective accounts gleaned only through participant interviews [20].

Patient and Public Involvement

Two head and neck patient groups were consulted during development of the research question, study design and protocol development, but patients were not involved in data gathering and analysis.

Ethical Approval

All participants gave written informed consent to be interviewed. Observational data (MDT meeting and clinic) were audiorecorded, then the patient was approached, two to three days after the event, to consent for inclusion in the study. If they agreed to be included, then the recordings were transcribed word for word. If they refused, all data collected so far were securely destroyed. This consent procedure was developed to avoid approaching the patient on the day of the treatment decision when they were already being given a lot of information. Ethical approval was gained from the NHS Research Ethics Newcastle and North Tyneside 2

For peer review only

committee (reference 11/NE/0200) in September 2011 and all necessary local Research and Development governance permissions were obtained.

Sampling

Initial sampling aimed to recruit patients who had a treatment decision to make about their care, or where more than one treatment option was available to the patient. Concepts arising from the patient-derived data drove the subsequent data collection and analysis. A range of staff members who were part of the MDT were also recruited for interview. Purposive sampling [21] guided the sampling strategy to explore emerging concepts with data collection and analysis occurring in tandem. Cases were included which would test the concepts and themes which were emerging. For example, in the early cases, palliative options were often not discussed or offered in the clinic, and so patients were included who had options for treatment, one of which was palliative, were included. Concepts explored through sampling also included uncertainty, assessment of best and trust. Thus, further sampling was guided by the emerging analysis [22] and continued until a state of theoretical sufficiency [23] was achieved. This means that data collection ceases when sufficient or adequate depth of understanding has been reached; this allows for a greater number and breadth of concepts to be explored in this complex setting using multiple data collection techniques

Observations

Non-participant observations of 35 MDT meetings and 37 MDT outpatient clinics were conducted. Patient with a diagnosis of new or recurrent head and neck cancer whose treatment options were being discussed in the MDT were included. They were excluded if they did not understand written or spoken English, or they did not have the capacity to consent. The MDT meetings and clinics were all audio-recorded and transcribed verbatim.

For peer review only - http://bmjopen.bmj.com/site/about/guidelines.xhtml

Detailed field notes were also made at the time of observation, then transcribed immediately afterwards.

Interviews

Semi-structured interviews were conducted with patients and staff. The development of the interview guide was iterative; as data collection continued, the content of the guide evolved in order to explore emerging themes[24]. In particular, the interview guide evolved to explore concepts of uncertainty (and how it is communicated), conversations around and attitudes towards palliative care, trust (between members of the MDT and between doctor and patient) and risk communication (see supplemental file). Informal interviews with staff members of the MDT also took place and were incorporated into written field notes. Pseudonyms are used for reporting data throughout to protect the anonymity of respondents.

Analysis

The data were analysed by one researcher (DWH) and emerging analyses were discussed with CE and BH, following principles of constructivist grounded theory [21]. Only one coder was used because of the complexity of the multiple data sources during this ethnographic study. However emerging concepts and themes were discussed formally in the wider research team. All data sources (MDT meeting, clinic, informal and formal interviews) were analysed using the same coding framework. The codes used were conceptual, rather than descriptive, and labels were derived completely from the data, not pre-determined. Line by line coding produced an initial coding framework: the emerging analysis was used during axial coding to guide further sampling and further development of the coding framework. Hence coding was both inductive and deductive and when the coding framework was altered, all transcripts were re-coded. The coding was organised using the NVivo computer package.

For peer review only - http://bmjopen.bmj.com/site/about/guidelines.xhtml

Emerging findings (and 'memos') were formally discussed in the research team to develop the data analysis and guide subsequent analysis and data sampling

Results

The research was conducted in three head and neck cancer (HNC) centres in the north east of England. A total of 35 MDT meetings and 37 clinic appointments MDT meetings and clinics were observed for 30 patients (23 males and seven females, aged 38-87 years). Additionally 23 interviews were conducted with patients and nine interviews with MDT members (see table one). In all centres, the MDT meeting took place without the patient present and was attended by surgeons, oncologists, radiologists, pathologists, speech and language therapists, dieticians and administrative staff. Following the meeting, one surgeon met with the patient in clinic. Sometimes other members were present with the surgeon, and other times they were alone. If considering non-surgical options, the patient would meet an oncologist. Each MDT would discuss between 10 and 30 patients; the majority of these patients were then seen in the accompanying clinic

For peer review only

PATIENTS: Group 1	Centre	Age	Tumour site	Observation		Int 1	Int 2
				MDT	Clinic		
Patient 1	A	68	Pharynx	1	1	1	1
Patient 2	A	82	Pharynx	1	1	x	x
Patient 3	A	61	Parotid	1	1	x	x
Patient 4	A	71	Lip	1	1	x	x
Patient 5	A	54	Pharynx	1	1	1	x
Patient 6	A	80	Pharynx	1	1	x	x
Patient 7	A	72	Pinna	1	1	x	x
Patient 8	A	87	Pharynx	1	1	1	x
Patient 9	A	64	Larynx	1	1	1	x
Patient 10	A	61	Larynx	1	1	1	x
Patient 11	A	52	Pharynx	1	1	x	x
Patient 12	A	55	Pharynx	1	1	1	1
Patient 13	A	62	Larynx	1	1	1	x
Patient 14	B	73	Pharynx	3	1	1	x
Patient 15	B	49	Pharynx	1	1	1	x
Patient 16	B	52	Pharynx	1	1	1	x
Patient 17	B	63	Larynx	1	1	x	x
Patient 18	B	49	Larynx	1	1	x	x
Patient 19	B	73	Mouth	3	1	1	x
Patient 20	B	65	Larynx	1	1	1	x
Patient 21	B	57	Pharynx	1	1	x	x
Patient 22	B	63	Pharynx	1	2	1	1
Patient 23	C	69	Pharynx	1	2	1	x
Patient 24	C	81	Mandible	1	2	x	x
Patient 25	C	60	Pharynx	1	1	1	x
Patient 26	C	67	Pharynx	1	1	x	x
Patient 27	C	46	Pharynx	1	2	x	x
Patient 28	C	38	Larynx	1	1	x	x
Patient 29	C	70	Larynx	1	4	1	x
Patient 30	C	84	Larynx	1	1	x	x
**PATIENTS: Group 2 (interview only)**							
Patient 31	A	82	Pharynx				
Patient 32	A	57	Larynx				
Patient 33	A	52	Pharynx				
Patient 34	B	65	Larynx				
**STAFF (interview only)**		**Staff role**					
Mr Red	A	ENT surgeon					
Dr Orange	A	Oncologist					
Mr Surton	A	Maxillofacial surgeon					
Miss Salt	A	Speech and Language Therapist					
Tessa Darling	A	Clinical Nurse Specialist					
Mr Halifax	B	Maxillofacial surgeon					
Mr Blaydon	B	ENT surgeon					
Mr North	B	ENT surgeon					
Dr Goodier	C	Oncologist					

For peer review only - http://bmjopen.bmj.com/site/about/guidelines.xhtml

Table one: details of included participants

”Best” treatment

The MDT meeting discussion often tends towards debate on which treatment is “best” for a patient amongst the available options. In the following interview extract, a maxillofacial surgeon describes his view of the aim of the MDT discussion:

> [The team] need to leave the MDT [meeting] with the treatment options ….prioritised. So a rank order of [the] best treatment clinically – slightly irrespective of the patient’s wishes. From a clinical point of view to try and get best outcome, this would be our first, this would be our second, this would be third and fourth and fifth. Then you discuss it with the patient and say, “This is what we think.”

In this data extract, the surgeon clearly states his view that the aim of the MDT discussion is to decide the “clinically” best treatment for the patient and even goes as far to say that this could be “irrespective” of the patient’s wishes. Teams frequently conclude their discussion of treatment options in the MDT meeting with an agreement for the recommendation (ie the MDT’s perception of “best” treatment). This recommendation is to be delivered to the patient. In the following extract, the MDT members are discussing the merits of surgery (laser) vs radiotherapy

> Mr Black (ENT surgeon): I have a database of the [laser resections] I have done …. tonsil and soft palate tumours, and it’s just….it’s something we need to take notice of
>
> Mr Red (ENT surgeon): Yeah, I think we’ll need to, we’ll have to discuss it another time or we’ll take up the whole morning on one case. But, I think there are arguments for and against...
>
> Dr Orange (oncologist): I would suggest he has radiotherapy, because he will have a slightly better functional outcome, and he’s 80 and …because of his age, and because of the possibly better function….would you Dr Yellow?
>
> Dr Yellow (oncologist): Yes

For peer review only - http://bmjopen.bmj.com/site/about/guidelines.xhtml

> Mr Red: I think there is a consensus view of the MDT, would be for radiotherapy
>
> MR Black: OK
>
> (Observation, MDT meeting)

Although, during this discussion, options of radiotherapy and laser were available to the patient, the position of the MDT meeting was to provide a recommendation for radiotherapy. Here, we see the members of the MDT preparing their 'party line' which is to be delivered to the patient in clinic. This recommendation for "best treatment" is often conveyed to the patient on its own or in preference to other options

The "cycle of paternalism"

Anxious patients, faced with complex decisions can lead leads to patients endorsing the paternalistic approach as they are given little or no information about the available treatment choices and therefore tend to delegate responsibility of the decision to the clinician:

> Pt: You know, they're the doctors, they're the professional people. And I'm just Joe Bloggs off the street. ...For a lot of years, I was a steel erector. I wouldn't expect you as a doctor coming along and doing what I could do. Do I?
>
> Interviewer: You feel a decision should be the doctor's decision?
>
> Pt: Oh, definitely, without a doubt. It's got to be the doctor's decision. How could I make a decision like that?

Here, the patient delegates the decision to the MDT without question; he is allowing the MDT's assessment of 'best' treatment to act as the sole basis for a treatment decision. If decision delegation is accepted as the method by which MDTs convey and make decisions, a paternalistic decision making process results. In this model, the patient accepts that the MDT's assessment of 'best' (and hence the treatment recommendation) is appropriate. It creates a "cycle of paternalism" with grateful patients accepting firm recommendations from clinicians and clinicians reassured that they are doing their best for their patients.

For peer review only - http://bmjopen.bmj.com/site/about/guidelines.xhtml

Delivery of the MDT treatment recommendation

Firm MDT recommendations can sometimes place the clinician in a difficult position when discussing options. The following extract is the clinic appointment for patient six (the MDT meeting extract was included above). Here Mr Black (who favoured laser in the MDT meeting, referred to here as “surgery”) was delivering the MDT recommendation for radiotherapy to the patient:

> Mr Black: After a lot of discussion, the consensus…. would be to give you radiation therapy…. that was what we jointly decided. And we think with that treatment there is a very good chance of controlling your disease completely….
>
> Pt: Well, I’ll do as you say
>
> Daughter: So there’s no other operation, it would just be radiotherapy?
>
> Mr Black: We discussed this at length at the meeting…. and the majority of people… felt that to be frank, except for me, felt that radiation would be the way forward. And…. that’s what we are offering to you as first line treatment. Unless you have any reservations, then we can think about other options.
>
> Pt: I’ll do as you say….
>
> Daughter: Right. So he would have to come into hospital every day? He’s a really bad traveller…
>
> Pt: You know when I come here I get all tensed up and travelling….
>
> Mr Black: Really? Is it likely you may then stop the treatment midway for whatever reason, because that would backfire very badly.
>
> Pt: I wouldn’t do that
>
> Mr Black: I know you asked about the surgical option. I promised people I wouldn’t say anything. But it is feasible to take it out surgically, and there is an option available, but the consensus at the MDT was to go ahead with radiation. Unless, as a family or yourself very strongly object to it and feel that you can’t go ahead with that, then of course the surgical option is always there. But as a group we felt that the best way forward was to offer you radiation
>
> Pt: Well. I’ll go with you
>
> (Observation, MDT Clinic)

For peer review only - http://bmjopen.bmj.com/site/about/guidelines.xhtml

The final treatment decision was to deliver radiation, but the interaction above reveals the challenges of being tasked by the MDT to give a single recommendation when it is used in a decision discussion with a patient. Once new information was gleaned from the patient in clinic (being a “really bad traveller”) Mr Black struggled with how to deal with the recommendations: was it a rule to be followed? Here, the rigidity of the treatment recommendation acted as a barrier to an open discussion about the treatment options available to the patient and thus inhibited shared decision making.

Patient engagement with MDT recommendations

Modern clinical practice cannot assume that the sole role of the patient is the acceptance of a single firm treatment recommendations. Patient 10 was a 61 year old patient with an advanced cancer of his larynx. In the MDT it was decided that surgery (total laryngectomy) should be delivered as a single recommendation. Radiotherapy is available, carries a lower chance of cure, but allows him to retain his voice box. The following data are from his clinic appointment:

> Mr Black (ENT surgeon): This tumour in your throat is a fairly big tumour, and it’s spread to the neck as well. We believe that there are two possible ways that we can manage this. At some parts of the scan, there is evidence that the tumour may have gone into the Adam’s apple cartilage…. If that is the case, surgery would be the only option to get rid of the tumour. But surgery would involve you losing your voicebox, losing part of the swallowing passage, you would need a big neck operation….Once we do the surgery, your speech will be different, you won’t be speaking the same. You will have a hole in the centre of your neck, a tracheostomy
>
> Pt: Nah, nah [shakes head]
>
> Mr Black: You wouldn’t fancy that?
>
> Pt: No
>
> Mr Black: That’s the surgical option. On the other side is the option of radiation therapy
>
> Pt: I would rather take a chance with that

For peer review only - http://bmjopen.bmj.com/site/about/guidelines.xhtml

(Observation, MDT Clinic)

Here, and throughout the course of this consultation, the patient made a decision to reject surgery, which reduces his length of survival from his cancer in order to preserve his voice box. He was adamant he did not want a complete removal of the voice box and part of the throat (pharygolaryngectomy) and the decision was eventually made to use radiotherapy. However, in the subsequent interview, patient 10 discussed the rationale for his decision:

> Pt: Well you see my mother died of cancer… my father died of cancer, and I've seen the way cancer works. I'm not being cheeky…. once they cut you open, it's like your letting fresh air into a bulb, it then just spreads, and they stitch you back up again and "We've cured it", right, for how long? And then it comes back again…
>
> Interviewer: And what's important to you when you're making that decision?
>
> Pt: Surviving as long as I can…, I mean if you get the year, 18 months it's better than getting two weeks isn't it?

His consultation, which was limited in exploring what mattered to him, leads to a decision that is potentially at odds with his aspirations revealed above. His aim of treatment (survival) is not matched by the actual treatment decision (radiotherapy). This patient perspective could not be incorporated into the previous MDT discussion (which happened before the clinic appointment), but equally the subsequent clinic appointment did not explore his preferences and what underpinned them, risking a treatment decision at odds with his preferences and values. If Mr Black had explored the options for treatment with the patient more, this mismatch of treatment preferences and values could have been identified, and perhaps deconstructed. Such information about values and preferences is essential to good shared decision making, however very difficult to incorporate into the MDT decision making structure.

For peer review only

Discussion

This study has found that patient engagement with the outcome of an MDT discussion (a recommendation for “best” treatment) is problematic. Often patients accept this recommendation in the clinic (perhaps precisely because it is presented as the “best” treatment). However, this acquiescence may be due to the disempowered position in which patients find themselves as they confront a terrifying diagnosis and a myriad of complex decision options. In turn, clinicians often view the acceptance of an MDT recommendation as delegation of the decision by the patient to the clinician, an assumption which can promulgate a ‘cycle of paternalism’, where anxious patients have little real choice other than to accept the clear guidance offered by the expert team. However, limiting patient involvement to acceptance or rejection of a firm recommendation leads to decisions which are not in line with patient values and can not be considered patient-centred, shared decision making.

The rigidity of the MDT recommendation can act as a barrier to an open discussion of the available options. If the patient role is limited to either acceptance or refusal of a single recommendation, true engagement is impossible. A truncated discussion of a single MDT recommendation for treatment prohibits shared decision-making using the “three talk model” , as central to this model is a discussion of the options for treatment. A shared decision-making consultation allows the patient and clinician to explore the risks, benefits and consequences of a treatment alternatives; a move from initial to informed preferences; and exploration of patient values to reach a shared decision [25].

The structure of MDT working has not significantly changed since its inception in 1996. NHS patients rarely attend their MDT meetings, modern cancer care mandates that all

For peer review only - http://bmjopen.bmj.com/site/about/guidelines.xhtml

patients are discussed in this setting[26] and interventions to increase the number of patients discussed in an MDT are still sought after[27].

The MDT recommendation

If the MDT meeting and clinic follow a paternalistic pathway, the way in which their recommendation is used is clear: it is delivered to the patient with an assumption that it will be accepted. In the paternalistic tradition, physicians are considered to be best placed to evaluate the trade-offs and pitfalls of treatment, and applied these to the decision process based on their evaluation of the best interests of the patient [28]. However, often in cancer care (particularly head and neck cancer), treatment options are available for a patient: which of these is “best” depends on the value you apply to the various aspects of the treatment. For example, is the priority of treatment cure or preservation of quality of life? What functional impact will a patient endure to achieve tumour control? What aspects of functional decline (such as speech, swallow or aesthetics) are most important? The answers to these questions are based on values: clinicians and patient do not share values [29-31]. Thus MDTs must ensure that treatment decisions are driven by patient values. Although patients may justifiably actively delegate some or all of the responsibility for the decision to the MDT members, at the same time, the MDT have a duty to ensure that this is not due to disempowerment or lack of access to the information required to take an active part in decision making. Hence the clinician has a role to, at the very least, support the patient to understand what is important to them before accepting the role as decision maker on the patient’s behalf

Outwith the MDT decision process, a treatment recommendation from an individual clinician can be modified depending on the ongoing interaction with the patient and the preferences expressed. An MDT recommendation, on the other hand, is problematic for MDT members who attempt to combine it with the values or preferences of the patient. Is it set in stone, an

For peer review only - http://bmjopen.bmj.com/site/about/guidelines.xhtml

obligatory 'best' which must be adhered to? If the patient disagrees with the recommendation, what action should the MDT member take? In this way, MDT recommendations are inflexible, especially in the light of new information from the patient which was not clear or known in the MDT meeting. In other words, information about values and preferences are vital to a shared decision but difficult to incorporate into the MDT decision making structure. As we have previously described [18], MDTs often build the "evidential patient" in the MDT meeting discussion. This may include information about a patient's values and preferences, but these are impossible to incorporate into a meeting discussion without the patient present and without making assumptions about the patient.

Modernising MDT decision-making

If we are to modernise the MDT decision making structure to improve patient involvement, the role of the MDT discussion and the structure of the clinic must recognise that patients often "distribute" decisions. Rapley [32] describes how patients demonstrate a 'relational autonomy' by distributing their decision amongst people, encounters, places and information sources. Promoting relational autonomy means that involving patients in decisions requires more than presenting options and awaiting a verdict, instead emphasising the importance of the interaction with the clinician, encouraging questions, correcting misunderstanding, constructing preferences and allowing disagreement [33]. Indeed, the MDT decision-making structure gives ample opportunity for MDT members to distribute their decision amongst colleagues, but does not afford the same opportunity to patients

If the patient is to be a true participant in shared decision making, an alternative model of MDT decision making is required. Some teams have explored the idea of a patient attending their own MDT meeting, with many patients reporting a positive experience[34]: this idea is popular amongst patient advocates [35], but clinicians have mixed views [7 35 36]. Small studies

For peer review only - http://bmjopen.bmj.com/site/about/guidelines.xhtml

have concluded that patients attending their own MDT allows for better information giving [37] [38]and the opportunity to ask questions and contribute information such as preference [39]; however included patients may have higher health literacy[40] raising the possibility that including patients has potential to widen health inequality. MDT members often feel that patients attending their own meeting would inhibit the discussion and cause patient anxiety [35]; relationships within the MDT are often longstanding with pre existing hierarchies which can present barriers to new user integration[41]Nevertheless, if patients are to be included in MDT meetings, clarity is required on how patients, their supporters and healthcare teams are supported to make it a positive and worthwhile experience[42].

Of key importance is that the MDT meeting is not a discussion of which option is 'best' for a particular patient, but should instead aim to determine which valid treatment options are available. In particular, palliative options (or options of 'doing nothing') are often inadequately explored [43]. Clinic structures should be flexible to allow patients to distribute their decision-making amongst information sources and people. The patient may be enabled to come to the initial consultation more informed and prepared for the discussion. There may be a role for pre MDT clinic with the patient meeting a surgeon, oncologist or specialist nurse, or a post MDT clinic to convey options and explore values and preferences, maybe with more than one clinician. The MDT meeting may take place in a small 'combined clinic' setting around the interaction with the patient. The MDT members provide support, resources and personnel to discuss the treatment options, communicate the risk and uncertainty, elicit values and explore them; a decision aid may support this work [44]. The team may consider providing an individual who is independent of the clinical team to act as a decision coach or navigator [45]. MDT members should be encouraged to update their training in supporting patients in shared decision making, consent and communication. This study provides a novel and rich account of the difficulties that patients face when making a decision in the context of

For peer review only - http://bmjopen.bmj.com/site/about/guidelines.xhtml

an MDT. MDT decision making is mandated internationally however the specific structure of the decision process varies widely. Although the structure presented here (MDT meeting without a patient present, recommendation delivered to the patient separately) is common, other models of MDT decision making may not face similar challenges. Also, ethnographic methods, in providing depth to explore a smaller number of concepts in more detail, may lack the breadth of findings to make this piece of work widely applicable. Nevertheless, whilst the setting may not be universally generalisable, we hope that the emergent conclusions will be.

It is time for the development and design of alternative models of team decision making which have a central role for the patient. Further work to develop new model of delivering team decision-making would be multifactorial, incorporating the development of the structure of the MDT meeting and clinic, support and training for MDT members and patients and the development of tools to be used in combination with team decisions. Qualitative approaches should explore stakeholders' views of intervention components, which should be co-designed with patients. Evaluation of such interventions requires novel trial design, comparing methods of decision making and evaluating decision quality. MDT decision making is now ubiquitous and therefore the urgent need of reform to meet the principles of shared decision making should be a priority for clinical teams and cancer researchers.

Author Contributions

DW Hamilton
Protocol development, ethical approval, data gathering, data analysis, manuscript preparation

B Heaven
Protocol development, ethical approval, data analysis, manuscript preparation

R G Thomson
Protocol development, data analysis, manuscript preparation

JA Wilson

For peer review only - http://bmjopen.bmj.com/site/about/guidelines.xhtml

Protocol development, data analysis, manuscript preparation

C Exley
Protocol development, ethical approval, data analysis, manuscript preparation

Competing interests

All authors have completed the Unified Competing Interest form at www.icmje.org/coi_disclosure.pdf (available on request from the corresponding author) and declare that DWH, BH, RT, JAW and CE have no relationships with any companies that might have an interest in the submitted work in the previous 3 years; and DWH, BH, RT, JAW and CE have no non-financial interests that may be relevant to the submitted work

Funding

This study represents independent research funded by the National Institute of Health Research. The views expressed are those of the author(s) and not necessarily those of the National Health Service, the National Institute of Health Research or the Department of Health

Data sharing statement

No additional data available

References

1. Licitra L, Keilholz U, Tahara M, et al. Evaluation of the benefit and use of multidisciplinary teams in the treatment of head and neck cancer. *Oral Oncol* 2016;59:73-9. doi: 10.1016/j.oraloncology.2016.06.002

2. Calman KC, Hine D. A Policy Framework for Commissioning Cancer Services In: Health Do, ed., 1995.

3. Kelly SL, Jackson JE, Hickey BE, et al. Multidisciplinary clinic care improves adherence to best practice in head and neck cancer. *American journal of otolaryngology* 2013;34(1):57-60. doi: 10.1016/j.amjoto.2012.08.010

4. Patil RD, Meinzen-Derr JK, Hendricks BL, et al. Improving access and timeliness of care for veterans with head and neck squamous cell carcinoma: A multidisciplinary team's approach. *Laryngoscope* 2016;126(3):627-31. doi: 10.1002/lary.25528

5. Starmer H, Sanguineti G, Marur S, et al. Multidisciplinary head and neck cancer clinic and adherence with speech pathology. *Laryngoscope* 2011;121(10):2131-5. doi: 10.1002/lary.21746

6. Taylor C, Munro AJ, Glynne-Jones R, et al. Multidisciplinary team working in cancer: what is the evidence? *Bmj* 2010;340:c951. doi: 10.1136/bmj.c951

For peer review only - http://bmjopen.bmj.com/site/about/guidelines.xhtml

For peer review only

7. NCAT. Multidisciplinary team members' views about MDT working: results from a survey commissioned by the National Cancer Action Team. London Kings College, 2009.

8. Rajan S, Foreman J, Wallis MG, et al. Multidisciplinary decisions in breast cancer: does the patient receive what the team has recommended? *British journal of cancer* 2013;108(12):2442-7. doi: 10.1038/bjc.2013.267

9. Blazeby JM, Wilson L, Metcalfe C, et al. Analysis of clinical decision-making in multi-disciplinary cancer teams. *Annals of oncology : official journal of the European Society for Medical Oncology / ESMO* 2006;17(3):457-60. doi: 10.1093/annonc/mdj102

10. Wood JJ, Metcalfe C, Paes A, et al. An evaluation of treatment decisions at a colorectal cancer multi-disciplinary team. *Colorectal disease : the official journal of the Association of Coloproctology of Great Britain and Ireland* 2008;10(8):769-72. doi: 10.1111/j.1463-1318.2007.01464.x

11. Taylor C, Atkins L, Richardson A, et al. Measuring the quality of MDT working: an observational approach. *BMC cancer* 2012;12:202. doi: 10.1186/1471-2407-12-202

12. Rowlands S, Callen J. A qualitative analysis of communication between members of a hospital-based multidisciplinary lung cancer team. *Eur J Cancer Care* 2013;22(1):20-31. doi: 10.1111/ecc.12004

13. Lanceley A, Savage J, Menon U, et al. Influences on multidisciplinary team decision-making. *Int J Gynecol Cancer* 2008;18(2):215-22. doi: DOI 10.1111/j.1525-1438.2007.00991.x

14. Soukup T, Murtagh G, Lamb BW, et al. Degrees of Multidisciplinarity Underpinning Care Planning for Patients with Cancer in Weekly Multidisciplinary Team Meetings: Conversation Analysis. *J Multidiscip Healthc* 2021;14:411-24. doi: 10.2147/JMDH.S270394 [published Online First: 2021/02/27]

15. Kidger J, Murdoch J, Donovan JL, et al. Clinical decision-making in a multidisciplinary gynaecological cancer team: a qualitative study. *Bjog-Int J Obstet Gy* 2009;116(4):511-17. doi: DOI 10.1111/j.1471-0528.2008.02066.x

16. Horlait M, Baes S, Dhaene S, et al. How multidisciplinary are multidisciplinary team meetings in cancer care? An observational study in oncology departments in Flanders, Belgium. *J Multidiscip Healthc* 2019;12:159-67. doi: 10.2147/JMDH.S196660 [published Online First: 2019/03/14]

17. Horlait M, De Regge M, Baes S, et al. Exploring non-physician care professionals' roles in cancer multidisciplinary team meetings: A qualitative study. *PLoS One* 2022;17(2):e0263611. doi: 10.1371/journal.pone.0263611 [published Online First: 2022/02/04]

18. Hamilton DW, Heaven B, Thomson RG, et al. Multidisciplinary team decision-making in cancer and the absent patient: a qualitative study. *BMJ Open* 2016;6(7):e012559. doi: 10.1136/bmjopen-2016-012559 [published Online First: 2016/07/23]

For peer review only - http://bmjopen.bmj.com/site/about/guidelines.xhtml

19. Taylor C, Finnegan-John J, Green JS. "No decision about me without me" in the context of cancer multidisciplinary team meetings: a qualitative interview study. *BMC health services research* 2014;14:488. doi: 10.1186/s12913-014-0488-2

20. Strauss AL. Continual permutations of action. New York: Aldine de Gruyter 1993.

21. Charmaz K, editor. *Constructing Grounded Theory. A Practical Guide Through Qualitative Analysis*. London: SAGE, 2006.

22. Strauss AL, Corbin JM. Basics of qualitative research : grounded theory procedures and techniques. Newbury Park, Calif.: Sage Publications 1990.

23. Dey I. Grounding grounded theory : guidelines for qualitative inquiry. San Diego: Academic Press 1999.

24. Green J, Thorogood N. Qualitative methods for health research. 2nd ed. Los Angeles: SAGE 2009.

25. Elwyn G, Durand MA, Song J, et al. A three-talk model for shared decision making: multistage consultation process. *Bmj* 2017;359:j4891. doi: 10.1136/bmj.j4891 [published Online First: 2017/11/08]

26. NICE. Improving Outcomes in Head and Neck Cancer - the Manual. London, 2004.

27. Maharaj AD, Evans SM, Zalcberg JR, et al. Barriers and enablers to the implementation of multidisciplinary team meetings: a qualitative study using the theoretical domains framework. *BMJ quality & safety* 2021;30(10):792-803. doi: 10.1136/bmjqs-2020-011793 [published Online First: 2020/11/29]

28. Charles C, Gafni A, Whelan T. Decision-making in the physician-patient encounter: revisiting the shared treatment decision-making model. *Social science & medicine* 1999;49(5):651-61. doi: S0277953699001458 [pii] [published Online First: 1999/08/19]

29. Hamilton DW, Bins JE, McMeekin P, et al. Quantity vs quality of life in laryngeal cancer: a time trade off study. *Clinical Otolarygology* 2012;37(Suppl. 1):108.

30. Hamilton DW, Pedersen A, Blanchford H, et al. A comparison of attitudes to laryngeal cancer treatment outcomes: A time trade-off study. *Clinical Otolaryngology* 2018;43(1):117-23. doi: 10.1111/coa.12906

31. Slevin ML, Stubbs L, Plant HJ, et al. Attitudes to chemotherapy: comparing views of patients with cancer with those of doctors, nurses, and general public. *Bmj* 1990;300(6737):1458-60. [published Online First: 1990/06/02]

32. Rapley T. Distributed decision making: the anatomy of decisions-in-action. *Sociol Health Illn* 2008;30(3):429-44. doi: SHIL1064 [pii]

10.1111/j.1467-9566.2007.01064.x [published Online First: 2008/01/16]

For peer review only - http://bmjopen.bmj.com/site/about/guidelines.xhtml

33. Entwistle VA, Carter SM, Cribb A, et al. Supporting patient autonomy: the importance of clinician-patient relationships. *J Gen Intern Med* 2010;25(7):741-5. doi: 10.1007/s11606-010-1292-2

34. Diekmann A, Heuser C, Ernstmann N, et al. How do breast cancer patients experience multidisciplinary tumor conferences? - A description from the patient perspective. *Breast* 2019;44:66-72. doi: 10.1016/j.breast.2018.12.012 [published Online First: 2019/01/23]

35. Butow P, Harrison JD, Choy ET, et al. Health professional and consumer views on involving breast cancer patients in the multidisciplinary discussion of their disease and treatment plan. *Cancer* 2007;110(9):1937-44. doi: 10.1002/cncr.23007

36. Heuser C, Diekmann A, Schellenberger B, et al. Patient Participation in Multidisciplinary Tumor Conferences from the Providers' Perspective: Is It Feasible in Routine Cancer Care? *J Multidiscip Healthc* 2020;13:1729-39. doi: 10.2147/JMDH.S283166 [published Online First: 2020/12/05]

37. Bellardita L, Donegani S, Spatuzzi AL, et al. Multidisciplinary Versus One-on-One Setting: A Qualitative Study of Clinicians' Perceptions of Their Relationship With Patients With Prostate Cancer. *Journal of oncology practice / American Society of Clinical Oncology* 2011;7(1):e1-5. doi: 10.1200/JOP.2010.000020

38. Choy ET, Chiu A, Butow P, et al. A pilot study to evaluate the impact of involving breast cancer patients in the multidisciplinary discussion of their disease and treatment plan. *Breast* 2007;16(2):178-89. doi: 10.1016/j.breast.2006.10.002

39. Bohmeier B, Schellenberger B, Diekmann A, et al. Opportunities and limitations of shared decision making in multidisciplinary tumor conferences with patient participation - A qualitative interview study with providers. *Patient education and counseling* 2021;104(4):792-99. doi: 10.1016/j.pec.2020.09.007 [published Online First: 2020/10/15]

40. Heuser C, Diekmann A, Kowalski C, et al. Health literacy and patient participation in multidisciplinary tumor conferences in breast cancer care: a multilevel modeling approach. *BMC cancer* 2019;19(1):330. doi: 10.1186/s12885-019-5546-z [published Online First: 2019/04/10]

41. Martin GP, Finn R. Patients as team members: opportunities, challenges and paradoxes of including patients in multi-professional healthcare teams. *Sociol Health Illn* 2011;33(7):1050-65. doi: 10.1111/j.1467-9566.2011.01356.x

42. Schellenberger B, Heuser C, Diekmann A, et al. Questions and emotional expressions from patients and companions while participating in multidisciplinary tumor conferences in breast and gynecological cancer centers. *Patient education and counseling* 2021 doi: 10.1016/j.pec.2021.12.010 [published Online First: 2021/12/27]

43. Nelson A, Longo M, Byrne A, et al. Chemotherapy decision-making in advanced lung cancer: a prospective qualitative study. *BMJ Support Palliat Care* 2020 doi: 10.1136/bmjspcare-2020-002395 [published Online First: 2020/08/23]

For peer review only - http://bmjopen.bmj.com/site/about/guidelines.xhtml

44. Stacey D, Legare F, Lewis K, et al. Decision aids for people facing health treatment or screening decisions. *Cochrane Db Syst Rev* 2017(4) doi: ARTN CD001431

10.1002/14651858.CD001431.pub5

45. Stacey D, Murray MA, Legare F, et al. Decision coaching to support shared decision making: a framework, evidence, and implications for nursing practice, education, and policy. *Worldviews Evid Based Nurs* 2008;5(1):25-35. doi: WVN108 [pii]

10.1111/j.1741-6787.2007.00108.x [published Online First: 2008/02/13]

For peer review only

For peer review only - http://bmjopen.bmj.com/site/about/guidelines.xhtml

Staff Interview Schedule

Introduce researcher and purpose of the study

Obtain consent to proceed and to record the conversation

- What do you see as the best way of making decisions in head and neck cancer?
- The MDT
    - What is it for? What is its primary aim?
    - What works well, and what works not so well?
    - How could it be improved?
    - No decision about me without me in the MDT…. Is this possible?
- Treatment decision
    - Why do you regard a treatment as the best for a patient?
    - What factors do you take into account when making treatment decisions?
- Patient involvement in decisions/shared decision making
    - What does it mean to you?
    - Do you think there are barriers to patient involvement? What are they? Why do they exist?
    - Why do clinicians struggle to involve their patients effectively?
    - Do you think we need to improve patient involvement? Why?
- Uncertainty/conflict
    - Is it a problem or is it healthy/required?
    - How should uncertainty and conflict be presented to the patient? Should they know that you don't know?
    - If there are options, how should they be communicated?
- Decision for treatment
    - What is the role of the patient in the treatment decision?
    - How much of a role should the patient have? Can patients know enough?
    - How much of a role should a patient have in the decision
        - Should we give power of vito?
        - Should you allow a patient to make a decision which is considered wrong?
        - how much should a patient know before treatment
    - How much of a role should the family have
- Palliation/prognosis
    - Do we palliate enough? Do we treat too many people radically?
    - What are the barriers to good palliation?
    - Do you think we have a range of palliative options available to us?
    - Do you have prognostic information available to you?
    - Would you use this information if it was available? How would you use it?

New patient interview 1 schedule

Introduce researcher and purpose of the study

Obtain consent to proceed and to record the conversation

The interview will cover the following broad areas:

- Previous experience of making healthcare decisions
    - What does shared decision making mean?
    - Have you had to make big healthcare decisions before?
- Experience of making this decision
    - What was your process of making the decision? Talk me though it
    - What did you draw upon?
    - What factors did you take into account?
    - Did you turn to anybody/anything for information or help?
    - How did you come to your final decision?
    - What was the MDT like?
    - Did you understand what was going on?
    - Could it be improved?
- Information given about the disease and treatment options
    - Do you think you had enough information?
    - Where do you get your information from?
    - Do you think you need more information?
    - Did you understand all the information
- Patient involvement in the decision about care
    - Should patients be involved in decisions about their care?
    - How should we involve patients?
    - Were you involved? How?
    - Would you have liked more say/less say?
    - How this made the participant feel, and further exploration
- What are your expectations of treatment?
    - Are you expecting side effects? What sort?
    - Do you have any idea of your prognosis? Do you want to know?
    - Where will you be in 6 months time?
- In the perfect world, how do you think decisions should be made?

For peer review only

New patient interview 2 schedule/retrospective patient interview schedule

Introduce researcher and purpose of the study

Obtain consent to proceed and to record the conversation

- Experience of the treatment
  - What has happened so far, story of the treatment
  - Correlation with expectations – worse, better
- New normal
  - Do you have a good quality of life?
  - How do you cope with swallowing?
  - How is your voice, how do you get on with communication?
- MDT and decision process
  - When you look back did you understand the treatment decision?
  - Did you understand the consequences of the decision?
  - Did you have enough information to base the decision on?
  - Should they have allowed you more or less control over the decision?
  - Do you wish the decision had been made differently?
  - Do you feel you made the right or the wrong decision?
  - Do you have any regrets?
  - How could they improve the clinic/the decision process?
  - In the perfect world, how do you think treatment decisions should be made?
- Palliation/prognosis
  - Were you ever aware of the chances of the treatment being successful?
  - Would you want to be aware?
  - Was palliation ever an option for you? Is it now?
- Information giving
  - Do you think you had enough information about treatment?
  - Did you understand what you were entering yourself into?
  - Should they give more information? Could they?

For peer review only

Topic	Page	Short description
1: Title	1	How do patients make decisions in the context of a multidisciplinary team: an ethnographic study of four head and neck cancer centres
2: Abstract	2-3	Included in manuscript
3: Problem formulation	4-5	MDT working is common and expensive, but the impact on shared decision making and patient involvement in decisions remains relatively unexplored
4: Purpose or research question	4-5	To examine critically patient engagement in the MDT treatment decision making process in head and neck cancer, and to evaluate the experience and practice of decision-making by patients and clinicians
5: Qualitative approach and research paradigm	5-7	Ethnographic methods (non-participant observation and semi-structured interviews). Analysis followed principles of constructivist grounded theory
6 Researcher characteristics and reflexivity	5	All data were collected by the lead author (DWH) whilst performing his PhD. At the time, he was a trainee surgeon and had taken time out of programme for research. The dual role of surgical trainee and researcher allowed easy access to the setting and language, making ethnography and observations easier and potentially more 'natural'. Co-authors BH and CE were involved in analysis in order to minimise the effect of the researcher's status on the emerging conclusions
7 Context	6&7	The research was conducted in four head and neck cancer centres in the north east of England. In all centres, the MDT meeting took place without the patient present; following this, one or more members of the MDT met with the patient in clinic
8 Sampling strategy	7	Initial sampling aimed to recruit patients who had a treatment decision to make about their care, or where more than one treatment option was available to the patient. . Purposive sampling guided the sampling strategy to explore emerging concepts with data collection and analysis occurring in tandem. Thus, further sampling was guided by the emerging analysis and continued until a state of theoretical sufficiency was achieved.
9 Ethical issues pertaining to human subjects	5	Ethical approval was gained from the NHS Research Ethics Newcastle and North Tyneside 2 committee (reference 11/NE/0200) in September 2011 and all necessary local Research and Development governance permissions were obtained. All participants provided informed consent.
10 Data collection methods	6	Non-participant observation; semi structured interviews with patients and staff; field notes; reflective notes. Iterative process with emerging themes explored through further data collection. All

For peer review only

		emerging analysis and memos discussed with co-authors (CE and BH)
11 Data collection instruments and technologies	6	Interview guides (iteratively developed) used for interviews. Field notes and notes from informal discussions. Audiorecording and word-for-word transcription of all formal interviews, MDT meeting and MDT clinic
12 Units of study	6	34 observations of MDT meetings and 37 clinic appointments (for 30 patients; 13 of these patients were interviewed once, three were interviewed twice). Interviews with four further treated patients and nine members of staff.
13 Data processing	6-7	All audiorecordings were transcribed word for word and anonymised. All reflective notes and field notes were anonymised. Patients and staff given pseudonyms. MDT meeting data transcribed by the main author (DWH) and all recording transcribed by others were checked by DWH. Audio recordings destroyed after transcription. Data kept on University computers, password protected
14 Data analysis	7	The data were analysed by one researcher (DWH) and emerging analyses were discussed with CE and BH, following principles of constructivist grounded theory. All data sources (MDT meeting, clinic, informal and formal interviews) were analysed using the same coding framework. Line by line coding produced an initial coding framework: the emerging analysis was used during axial coding to guide further sampling and further development of the coding framework; when the coding framework was altered, all transcripts were re-coded. The codes used were conceptual, rather than descriptive, and labels were derived completely from the data, not pre-determined. The coding was organised using the NVivo computer package. Emerging findings (and 'memos') were discussed in the research team (particularly with CE and BH) to develop the data analysis and guide subsequent analysis and data sampling
15 Techniques to enhance trustworthiness	7	All emerging themes and analysis were discussed in the research team with CE and BH involved in analysis. Findings presented in departmental meetings
16 Synthesis and interpretation	7-12	The outcome of an MDT discussion is frequently a "in the clinic as they confront a terrifying diagnosis and a myriad of complex decision options. In turn,.
17 Links to empirical data	7-12	Data presented in the results section linked to empirical data throughout
18 Integration with prior work, implications, transferability and contributions to the field	12-16	The discussion section discusses the findings in the context of prior work and the contributions to the field

For peer review only

19 Limitations	3, 18-19	Strengths and limitations are discussed in the discussion section and there is a list of strengths and limitations at the beginning of the manuscript
20 Conflict of interest	16	All authors have completed the Unified Competing Interest form at www.icmje.org/coi_disclosure.pdf (available on request from the corresponding author) and declare that DWH, BH, RT, JAW and CE have no relationships with any companies that might have an interest in the submitted work in the previous 3 years; and DWH, BH, RT, JAW and CE have no non-financial interests that may be relevant to the submitted work
21 Funding	17	This study represents independent research funded by the National Institute of Health Research. The views expressed are those of the author(s) and not necessarily those of the National Health Service, the National Institute of Health Research or the Department of Health

For peer review only

BMJ Open

How do patients make decisions in the context of a multidisciplinary team: an ethnographic study of four head and neck cancer centres in the north of England

Journal:	*BMJ Open*
Manuscript ID	bmjopen-2022-061654.R3
Article Type:	Original research
Date Submitted by the Author:	20-Jul-2022
Complete List of Authors:	Hamilton, David; Newcastle University, Population Health Sciences Institute; Newcastle Upon Tyne Hospitals NHS Foundation Trust, ENT<br>Heaven, Benjamin; Newcastle University, Population Health Sciences Institute<br>Thomson, Richard; Newcastle University, Population Health Sciences Institute<br>Wilson, Janet; Newcastle University, Population Health Sciences Institute<br>Exley, Catherine; Newcastle University, Population Health Sciences Institute
**Primary Subject Heading**:	Patient-centred medicine
Secondary Subject Heading:	Communication, Ear, nose and throat/otolaryngology, Health services research, Qualitative research
Keywords:	Organisation of health services < HEALTH SERVICES ADMINISTRATION & MANAGEMENT, Quality in health care < HEALTH SERVICES ADMINISTRATION & MANAGEMENT, Head & neck tumours < ONCOLOGY, Head & neck surgery < OTOLARYNGOLOGY, QUALITATIVE RESEARCH

SCHOLARONE™
Manuscripts

I, the Submitting Author has the right to grant and does grant on behalf of all authors of the Work (as defined in the below author licence), an exclusive licence and/or a non-exclusive licence for contributions from authors who are: i) UK Crown employees; ii) where BMJ has agreed a CC-BY licence shall apply, and/or iii) in accordance with the terms applicable for US Federal Government officers or employees acting as part of their official duties; on a worldwide, perpetual, irrevocable, royalty-free basis to BMJ Publishing Group Ltd ("BMJ") its licensees and where the relevant Journal is co-owned by BMJ to the co-owners of the Journal, to publish the Work in this journal and any other BMJ products and to exploit all rights, as set out in our licence.

The Submitting Author accepts and understands that any supply made under these terms is made by BMJ to the Submitting Author unless you are acting as an employee on behalf of your employer or a postgraduate student of an affiliated institution which is paying any applicable article publishing charge ("APC") for Open Access articles. Where the Submitting Author wishes to make the Work available on an Open Access basis (and intends to pay the relevant APC), the terms of reuse of such Open Access shall be governed by a Creative Commons licence – details of these licences and which Creative Commons licence will apply to this Work are set out in our licence referred to above.

Other than as permitted in any relevant BMJ Author's Self Archiving Policies, I confirm this Work has not been accepted for publication elsewhere, is not being considered for publication elsewhere and does not duplicate material already published. I confirm all authors consent to publication of this Work and authorise the granting of this licence.

For peer review only

Title: How do patients make decisions in the context of a multidisciplinary team: an ethnographic study of four head and neck cancer centres in the north of England

DW Hamilton
Consultant Otolaryngology, Head and Neck Surgery, Honorary Senior Lecturer
Freeman Hospital, High Heaton, Newcastle upon Tyne NE7 7DN
Population Health Sciences Institute, Newcastle University, Newcastle upon Tyne, NE2 4AX

B Heaven
Formerly Newcastle University, has now left academia

R G Thomson
Emeritus Professor
Population Health Sciences Institute, Newcastle University, Newcastle upon Tyne, NE2 4AX

JA Wilson
Emerita Professor of Otolaryngology, Head and Neck Surgery
Population Health Sciences Institute, Newcastle University, Newcastle upon Tyne, NE2 4AX

C Exley
Professor of Qualitative Health Research
Population Health Sciences Institute, Newcastle University, Newcastle upon Tyne, NE2 4AX

Corresponding Author:
DW Hamilton
Freeman Hospital, High Heaton, Newcastle upon Tyne NE7 7DN
David.hamilton@ncl.ac.uk

Keywords
Decision Making, Shared
Communication
Cancer
Anthropology, Cultural
Informed consent
Autonomy

For peer review only

Abstract

Objectives

To describe how patients are engaged with cancer decisions in the context of multidisciplinary team (MDT) and how MDT recommendations are operationalised in the context of a shared decision.

Design

Ethnographic qualitative study

Setting

Four head and neck cancer centres in the north of England

Participants

Patients with a diagnosis of new or recurrent head and neck cancer; non-participant observation of 35 MDT meetings and 37 MDT clinics, informal interviews, and formal, semi-structured interviews with 20 patients and 9 MDT staff members

Methods

Ethnographic methods including non-participant observation of MDT meetings and clinic appointments, informal interviews, field notes and formal semi-structured interviews with patients and MDT members

Results

MDT discussions often conclude with a firm recommendation for treatment. When delivered to a patient in clinic this recommendation is often accepted by the patient, but this response may result from the disempowered position in which they find themselves. Whilst patient behaviour may thus appear to endorse clinicians' views that a paternalistic approach is desired by patients (creating a "cycle of paternalism"), the rigidity of the MDT treatment

For peer review only - http://bmjopen.bmj.com/site/about/guidelines.xhtml

For peer review only

recommendation can act as a barrier to discussion of options and the exploration of patient values.

Conclusions

The current model of MDT decision making does not support shared decision making and may actively undermine it. A model should be developed whereby the individual patient perspective has more input into MDT discussions, and where decisions are made on potential treatment options rather than providing a single recommendation for discussion with the patient. Deeper consideration should be given to how the MDT incorporates the patient perspective and/or delivers its discussion of options to the patient. In order to achieve these objectives, a new model of MDT working is required.

Word count: 3732

Strengths and limitations of this study

- This ethnographic study provides an in-depth analysis of the complexities of patient involvement and interaction with MDT decision making
- The methods (direct observation and semi structured interviews) allow a rich, data-driven analysis of a complex decision-making environment
- Head and neck cancer involves the trade off of function for survival and is thus a useful model when exploring complex decision making
- As is commonplace in qualitative researcher one researcher led the sampling, collecting and analysis, but the whole team were involved in discussions about interpretation of the data
- Although the structure of MDT decision making discussed here predominates in the UK, the issues faced will not be applicable to all teams

For peer review only - http://bmjopen.bmj.com/site/about/guidelines.xhtml

Introduction

Multidisciplinary team (MDT) decision making is internationally mandated to support appropriate high-quality treatment of patients with cancer [1]. In the UK, MDT working was established following the Calman-Hine report [2] and improves many aspects of cancer treatment such as staging, recruitment to trials [1], adherence to treatment guidelines [3], use of effective evidence-based therapy, timeliness of care [4] and access to the allied members of the healthcare team [5]. However the practice is time consuming and expensive, costing at least £100 million a year in the UK for data preparation and the same amount again for attendance in the UK [6]. To date no MDT cost-benefit analysis has been performed.

MDT members report that consideration of the patient as a person in decision making as a vital part of the decision-making process. In a survey of 2054 MDT members, 95% of respondents felt that "Patient views should always inform the decision-making process" and "Patient views/preferences should be presented to the MDT by somebody who has met the patient" [7]. Omitting patient preference information has an effect on the implementation of MDT recommendations [8-10]. MDT meetings are often dominated by discussion among doctors rather than including other MDT members who may know the patient better or have a more patient-centred perspective [11-14] creating a predominance of the biomedical model of disease [15-17]. This means that the stated aim of many MDT members - to have the patient central to the MDT treatment discussion - is at odds with the reality of the MDT process

We have previously described that if MDT meetings are to become more patient-centred, merely introducing increasing amounts of information about the patient into the MDT is not sufficient [18]. Although we know that the direct viewpoint of the patient within the MDT is lacking [19], there is to date no account of how patients engage with decisions about their treatment in the context of MDTs. This work aims to explore the experience of making

For peer review only - http://bmjopen.bmj.com/site/about/guidelines.xhtml

decisions in the context of an MDT, with a particular emphasis on the patient experience of the decision process.

Methods

This qualitative study used non-participant observation and semi-structured interviews to critically examine how decisions were made in and around the MDT with a particular focus on patient centredness. All data were collected by one researcher (DWH), a head and neck surgeon. Non-participant observation enables the researcher to study participants in their natural environment, and adds value to retrospective accounts gleaned only through participant interviews [20].

Patient and Public Involvement

Two head and neck patient groups were consulted during development of the research question, study design and protocol development, but patients were not involved in data gathering and analysis.

Ethical Approval

All participants gave written informed consent to be interviewed. Observational data (MDT meeting and clinic) were audiorecorded, then the patient was approached, two to three days after the event, to consent for inclusion in the study. If they agreed to be included, then the recordings were transcribed word for word. If they refused, all data collected so far were securely destroyed. This consent procedure was developed to avoid approaching the patient on the day of the treatment decision when they were already being given a lot of information. Ethical approval was gained from the NHS Research Ethics Newcastle and North Tyneside 2

For peer review only - http://bmjopen.bmj.com/site/about/guidelines.xhtml

committee (reference 11/NE/0200) in September 2011 and all necessary local Research and Development governance permissions were obtained.

Sampling

Initial sampling aimed to recruit patients who had a treatment decision to make about their care, or where more than one treatment option was available to the patient. Concepts arising from the patient-derived data drove the subsequent data collection and analysis. A range of staff members who were part of the MDT were also recruited for interview. Purposive sampling [21] guided the sampling strategy to explore emerging concepts with data collection and analysis occurring in tandem. Cases were included which would test the concepts and themes which were emerging. For example, in the early cases, palliative options were often not discussed or offered in the clinic, and so patients were included who had options for treatment, one of which was palliative, were included. Concepts explored through sampling also included uncertainty, assessment of best and trust. Thus, further sampling was guided by the emerging analysis [22] and continued until a state of theoretical sufficiency [23] was achieved. This means that data collection ceases when sufficient or adequate depth of understanding has been reached; this allows for a greater number and breadth of concepts to be explored in this complex setting using multiple data collection techniques

For peer review only

Observations

Non-participant observations of 35 MDT meetings and 37 MDT outpatient clinics were conducted. Patient with a diagnosis of new or recurrent head and neck cancer whose treatment options were being discussed in the MDT were included. They were excluded if they did not understand written or spoken English, or they did not have the capacity to consent. The MDT meetings and clinics were all audio-recorded and transcribed verbatim.

For peer review only - http://bmjopen.bmj.com/site/about/guidelines.xhtml

Detailed field notes were also made at the time of observation, then transcribed immediately afterwards.

Interviews

Semi-structured interviews were conducted with patients and staff. The development of the interview guide was iterative; as data collection continued, the content of the guide evolved in order to explore emerging themes[24]. In particular, the interview guide evolved to explore concepts of uncertainty (and how it is communicated), conversations around and attitudes towards palliative care, trust (between members of the MDT and between doctor and patient) and risk communication (see supplemental file). Informal interviews with staff members of the MDT also took place and were incorporated into written field notes. Pseudonyms are used for reporting data throughout to protect the anonymity of respondents.

Analysis

The data were analysed by one researcher (DWH) and emerging analyses were discussed with CE and BH, following principles of constructivist grounded theory [21]. Only one coder was used because of the complexity of the multiple data sources during this ethnographic study. However emerging concepts and themes were discussed formally in the wider research team. All data sources (MDT meeting, clinic, informal and formal interviews) were analysed using the same coding framework. The codes used were conceptual, rather than descriptive, and labels were derived completely from the data, not pre-determined. Line by line coding produced an initial coding framework: the emerging analysis was used during axial coding to guide further sampling and further development of the coding framework. Hence coding was both inductive and deductive and when the coding framework was altered, all transcripts were re-coded. The coding was organised using the NVivo computer package.

For peer review only - http://bmjopen.bmj.com/site/about/guidelines.xhtml

Emerging findings (and 'memos') were formally discussed in the research team to develop the data analysis and guide subsequent analysis and data sampling

Results

The research was conducted in three head and neck cancer (HNC) centres in the north east of England. A total of 35 MDT meetings and 37 clinic appointments MDT meetings and clinics were observed for 30 patients (23 males and seven females, aged 38-87 years). Additionally 23 interviews were conducted with patients and nine interviews with MDT members (see table one). In all centres, the MDT meeting took place without the patient present and was attended by surgeons, oncologists, radiologists, pathologists, speech and language therapists, dieticians and administrative staff. Following the meeting, one surgeon met with the patient in clinic. Sometimes other members were present with the surgeon, and other times they were alone. If considering non-surgical options, the patient would meet an oncologist. Each MDT would discuss between 10 and 30 patients; the majority of these patients were then seen in the accompanying clinic

For peer review only

For peer review only - http://bmjopen.bmj.com/site/about/guidelines.xhtml

PATIENTS: Group 1	Centre	Age	Tumour site	Observation		Int 1	Int 2
				MDT	Clinic		
Patient 1	A	68	Pharynx	1	1	1	1
Patient 2	A	82	Pharynx	1	1	x	x
Patient 3	A	61	Parotid	1	1	x	x
Patient 4	A	71	Lip	1	1	x	x
Patient 5	A	54	Pharynx	1	1	1	x
Patient 6	A	80	Pharynx	1	1	x	x
Patient 7	A	72	Pinna	1	1	x	x
Patient 8	A	87	Pharynx	1	1	1	x
Patient 9	A	64	Larynx	1	1	1	x
Patient 10	A	61	Larynx	1	1	1	x
Patient 11	A	52	Pharynx	1	1	x	x
Patient 12	A	55	Pharynx	1	1	1	1
Patient 13	A	62	Larynx	1	1	1	x
Patient 14	B	73	Pharynx	3	1	1	x
Patient 15	B	49	Pharynx	1	1	1	x
Patient 16	B	52	Pharynx	1	1	1	x
Patient 17	B	63	Larynx	1	1	x	x
Patient 18	B	49	Larynx	1	1	x	x
Patient 19	B	73	Mouth	3	1	1	x
Patient 20	B	65	Larynx	1	1	1	x
Patient 21	B	57	Pharynx	1	1	x	x
Patient 22	B	63	Pharynx	1	2	1	1
Patient 23	C	69	Pharynx	1	2	1	x
Patient 24	C	81	Mandible	1	2	x	x
Patient 25	C	60	Pharynx	1	1	1	x
Patient 26	C	67	Pharynx	1	1	x	x
Patient 27	C	46	Pharynx	1	2	x	x
Patient 28	C	38	Larynx	1	1	x	x
Patient 29	C	70	Larynx	1	4	1	x
Patient 30	C	84	Larynx	1	1	x	x
**PATIENTS: Group 2 (interview only)**							
Patient 31	A	82	Pharynx				
Patient 32	A	57	Larynx				
Patient 33	A	52	Pharynx				
Patient 34	B	65	Larynx				
**STAFF (interview only)**		**Staff role**					
Staff 1	A	ENT surgeon					
Staff 2	A	Oncologist					
Staff 3	A	Maxillofacial surgeon					
Staff 4	A	Speech and Language Therapist					
Staff 5	A	Clinical Nurse Specialist					
Staff 6	B	Maxillofacial surgeon					
Staff 7	B	ENT surgeon					
Staff 8	B	ENT surgeon					
Staff 9	C	Oncologist					

For peer review only

For peer review only - http://bmjopen.bmj.com/site/about/guidelines.xhtml

Table one: details of included participants

"Best" treatment

The MDT meeting discussion often tends towards debate on which treatment is "best" for a patient amongst the available options. In the following interview extract, a maxillofacial surgeon describes his view of the aim of the MDT discussion:

> [The team] need to leave the MDT [meeting] with the treatment options ….prioritised. So a rank order of [the] best treatment clinically – slightly irrespective of the patient's wishes. From a clinical point of view to try and get best outcome, this would be our first, this would be our second, this would be third and fourth and fifth. Then you discuss it with the patient and say, "This is what we think."

In this data extract, the surgeon clearly states his view that the aim of the MDT discussion is to decide the "clinically" best treatment for the patient and even goes as far to say that this could be "irrespective" of the patient's wishes. Teams frequently conclude their discussion of treatment options in the MDT meeting with an agreement for the recommendation (ie the MDT's perception of "best" treatment). This recommendation is to be delivered to the patient. In the following extract, the MDT members are discussing the merits of surgery (laser) vs radiotherapy

> ENT surgeon 1: I have a database of the [laser resections] I have done …. tonsil and soft palate tumours, and it's just….it's something we need to take notice of
>
> ENT surgeon 2: Yeah, I think we'll need to, we'll have to discuss it another time or we'll take up the whole morning on one case. But, I think there are arguments for and against...
>
> Oncologist 1: I would suggest he has radiotherapy, because he will have a slightly better functional outcome, and he's 80 and …because of his age, and because of the possibly better function….would you Dr Yellow?
>
> Oncologist 2: Yes

For peer review only - http://bmjopen.bmj.com/site/about/guidelines.xhtml

ENT surgeon 2: I think there is a consensus view of the MDT, would be for radiotherapy

ENT surgeon 1: OK

(Observation, MDT meeting)

Although, during this discussion, options of radiotherapy and laser were available to the patient, the position of the MDT meeting was to provide a recommendation for radiotherapy. Here, we see the members of the MDT preparing their 'party line' which is to be delivered to the patient in clinic. This recommendation for "best treatment" is often conveyed to the patient on its own or in preference to other options

The "cycle of paternalism"

Anxious patients, faced with complex decisions can lead leads to patients endorsing the paternalistic approach as they are given little or no information about the available treatment choices and therefore tend to delegate responsibility of the decision to the clinician:

Pt: You know, they're the doctors, they're the professional people. And I'm just Joe Bloggs off the street. ...For a lot of years, I was a steel erector. I wouldn't expect you as a doctor coming along and doing what I could do. Do I?

Interviewer: You feel a decision should be the doctor's decision?

Pt: Oh, definitely, without a doubt. It's got to be the doctor's decision. How could I make a decision like that?

Here, the patient delegates the decision to the MDT without question; he is allowing the MDT's assessment of 'best' treatment to act as the sole basis for a treatment decision. If decision delegation is accepted as the method by which MDTs convey and make decisions, a paternalistic decision making process results. In this model, the patient accepts that the MDT's assessment of 'best' (and hence the treatment recommendation) is appropriate. It creates a "cycle of paternalism" with grateful patients accepting firm recommendations from clinicians and clinicians reassured that they are doing their best for their patients.

For peer review only - http://bmjopen.bmj.com/site/about/guidelines.xhtml

Delivery of the MDT treatment recommendation

Firm MDT recommendations can sometimes place the clinician in a difficult position when discussing options. The following extract is the clinic appointment for patient six (the MDT meeting extract was included above). Here the ENT surgeon (who favoured laser in the MDT meeting, referred to here as "surgery") was delivering the MDT recommendation for radiotherapy to the patient:

> ENT surgeon: After a lot of discussion, the consensus…. would be to give you radiation therapy…. that was what we jointly decided. And we think with that treatment there is a very good chance of controlling your disease completely….
>
> Pt: Well, I'll do as you say
>
> Daughter: So there's no other operation, it would just be radiotherapy?
>
> ENT surgeon: We discussed this at length at the meeting…. and the majority of people… felt that to be frank, except for me, felt that radiation would be the way forward. And…. that's what we are offering to you as first line treatment. Unless you have any reservations, then we can think about other options.
>
> Pt: I'll do as you say….
>
> Daughter: Right. So he would have to come into hospital every day? He's a really bad traveller…
>
> Pt: You know when I come here I get all tensed up and travelling….
>
> ENT surgeon: Really? Is it likely you may then stop the treatment midway for whatever reason, because that would backfire very badly.
>
> Pt: I wouldn't do that
>
> ENT surgeon: I know you asked about the surgical option. I promised people I wouldn't say anything. But it is feasible to take it out surgically, and there is an option available, but the consensus at the MDT was to go ahead with radiation. Unless, as a family or yourself very strongly object to it and feel that you can't go ahead with that, then of course the surgical option is always there. But as a group we felt that the best way forward was to offer you radiation
>
> Pt: Well. I'll go with you
>
> (Observation, MDT Clinic)

For peer review only - http://bmjopen.bmj.com/site/about/guidelines.xhtml

The final treatment decision was to deliver radiation, but the interaction above reveals the challenges of being tasked by the MDT to give a single recommendation when it is used in a decision discussion with a patient. Once new information was gleaned from the patient in clinic (being a “really bad traveller”) the surgeon struggled with how to deal with the recommendations: was it a rule to be followed? Here, the rigidity of the treatment recommendation acted as a barrier to an open discussion about the treatment options available to the patient and thus inhibited shared decision making.

Patient engagement with MDT recommendations

Modern clinical practice cannot assume that the sole role of the patient is the acceptance of a single firm treatment recommendations. Patient 10 was a 61 year old patient with an advanced cancer of his larynx. In the MDT it was decided that surgery (total laryngectomy) should be delivered as a single recommendation. Radiotherapy is available, carries a lower chance of cure, but allows him to retain his voice box. The following data are from his clinic appointment:

> ENT surgeon: This tumour in your throat is a fairly big tumour, and it’s spread to the neck as well. We believe that there are two possible ways that we can manage this. At some parts of the scan, there is evidence that the tumour may have gone into the Adam’s apple cartilage…. If that is the case, surgery would be the only option to get rid of the tumour. But surgery would involve you losing your voicebox, losing part of the swallowing passage, you would need a big neck operation….Once we do the surgery, your speech will be different, you won’t be speaking the same. You will have a hole in the centre of your neck, a tracheostomy
>
> Pt: Nah, nah [shakes head]
>
> ENT surgeon: You wouldn’t fancy that?
>
> Pt: No
>
> ENT surgeon: That’s the surgical option. On the other side is the option of radiation therapy
>
> Pt: I would rather take a chance with that

For peer review only - http://bmjopen.bmj.com/site/about/guidelines.xhtml

(Observation, MDT Clinic)

Here, and throughout the course of this consultation, the patient made a decision to reject surgery, which reduces his length of survival from his cancer in order to preserve his voice box. He was adamant he did not want a complete removal of the voice box and part of the throat (pharygolaryngectomy) and the decision was eventually made to use radiotherapy. However, in the subsequent interview, patient 10 discussed the rationale for his decision:

> Pt: Well you see my mother died of cancer… my father died of cancer, and I've seen the way cancer works. I'm not being cheeky…. once they cut you open, it's like your letting fresh air into a bulb, it then just spreads, and they stitch you back up again and "We've cured it", right, for how long? And then it comes back again…
>
> Interviewer: And what's important to you when you're making that decision?
>
> Pt: Surviving as long as I can…, I mean if you get the year, 18 months it's better than getting two weeks isn't it?

His consultation, which was limited in exploring what mattered to him, leads to a decision that is potentially at odds with his aspirations revealed above. His aim of treatment (survival) is not matched by the actual treatment decision (radiotherapy). This patient perspective could not be incorporated into the previous MDT discussion (which happened before the clinic appointment), but equally the subsequent clinic appointment did not explore his preferences and what underpinned them, risking a treatment decision at odds with his preferences and values. If the surgeon had explored the options for treatment with the patient more, this mismatch of treatment preferences and values could have been identified, and perhaps deconstructed. Such information about values and preferences is essential to good shared decision making, however very difficult to incorporate into the MDT decision making structure.

For peer review only - http://bmjopen.bmj.com/site/about/guidelines.xhtml

Discussion

This study has found that patient engagement with the outcome of an MDT discussion (a recommendation for “best” treatment) is problematic. Often patients accept this recommendation in the clinic (perhaps precisely because it is presented as the “best” treatment). However, this acquiescence may be due to the disempowered position in which patients find themselves as they confront a terrifying diagnosis and a myriad of complex decision options. In turn, clinicians often view the acceptance of an MDT recommendation as delegation of the decision by the patient to the clinician, an assumption which can promulgate a ‘cycle of paternalism’, where anxious patients have little real choice other than to accept the clear guidance offered by the expert team. However, limiting patient involvement to acceptance or rejection of a firm recommendation leads to decisions which are not in line with patient values and can not be considered patient-centred, shared decision making.

The rigidity of the MDT recommendation can act as a barrier to an open discussion of the available options. If the patient role is limited to either acceptance or refusal of a single recommendation, true engagement is impossible. A truncated discussion of a single MDT recommendation for treatment prohibits shared decision-making using the “three talk model” , as central to this model is a discussion of the options for treatment. A shared decision-making consultation allows the patient and clinician to explore the risks, benefits and consequences of a treatment alternatives; a move from initial to informed preferences; and exploration of patient values to reach a shared decision [25].

The structure of MDT working has not significantly changed since its inception in 1996. NHS patients rarely attend their MDT meetings, modern cancer care mandates that all

For peer review only - http://bmjopen.bmj.com/site/about/guidelines.xhtml

patients are discussed in this setting[26] and interventions to increase the number of patients discussed in an MDT are still sought after[27].

The MDT recommendation

If the MDT meeting and clinic follow a paternalistic pathway, the way in which their recommendation is used is clear: it is delivered to the patient with an assumption that it will be accepted. In the paternalistic tradition, physicians are considered to be best placed to evaluate the trade-offs and pitfalls of treatment, and applied these to the decision process based on their evaluation of the best interests of the patient [28]. However, often in cancer care (particularly head and neck cancer), treatment options are available for a patient: which of these is "best" depends on the value you apply to the various aspects of the treatment. For example, is the priority of treatment cure or preservation of quality of life? What functional impact will a patient endure to achieve tumour control? What aspects of functional decline (such as speech, swallow or aesthetics) are most important? The answers to these questions are based on values: clinicians and patient do not share values [29-31]. Thus MDTs must ensure that treatment decisions are driven by patient values. Although patients may justifiably actively delegate some or all of the responsibility for the decision to the MDT members, at the same time, the MDT have a duty to ensure that this is not due to disempowerment or lack of access to the information required to take an active part in decision making. Hence the clinician has a role to, at the very least, support the patient to understand what is important to them before accepting the role as decision maker on the patient's behalf

Outwith the MDT decision process, a treatment recommendation from an individual clinician can be modified depending on the ongoing interaction with the patient and the preferences expressed. An MDT recommendation, on the other hand, is problematic for MDT members who attempt to combine it with the values or preferences of the patient. Is it set in stone, an

For peer review only - http://bmjopen.bmj.com/site/about/guidelines.xhtml

obligatory 'best' which must be adhered to? If the patient disagrees with the recommendation, what action should the MDT member take? In this way, MDT recommendations are inflexible, especially in the light of new information from the patient which was not clear or known in the MDT meeting. In other words, information about values and preferences are vital to a shared decision but difficult to incorporate into the MDT decision making structure. As we have previously described [18], MDTs often build the "evidential patient" in the MDT meeting discussion. This may include information about a patient's values and preferences, but these are impossible to incorporate into a meeting discussion without the patient present and without making assumptions about the patient.

Modernising MDT decision-making

If we are to modernise the MDT decision making structure to improve patient involvement, the role of the MDT discussion and the structure of the clinic must recognise that patients often "distribute" decisions. Rapley [32] describes how patients demonstrate a 'relational autonomy' by distributing their decision amongst people, encounters, places and information sources. Promoting relational autonomy means that involving patients in decisions requires more than presenting options and awaiting a verdict, instead emphasising the importance of the interaction with the clinician, encouraging questions, correcting misunderstanding, constructing preferences and allowing disagreement [33]. Indeed, the MDT decision-making structure gives ample opportunity for MDT members to distribute their decision amongst colleagues, but does not afford the same opportunity to patients

If the patient is to be a true participant in shared decision making, an alternative model of MDT decision making is required. Some teams have explored the idea of a patient attending their own MDT meeting, with many patients reporting a positive experience[34]: this idea is popular amongst patient advocates [35], but clinicians have mixed views [7 35 36]. Small studies

For peer review only - http://bmjopen.bmj.com/site/about/guidelines.xhtml

have concluded that patients attending their own MDT allows for better information giving [37] [38]and the opportunity to ask questions and contribute information such as preference [39]; however included patients may have higher health literacy[40] raising the possibility that including patients has potential to widen health inequality. MDT members often feel that patients attending their own meeting would inhibit the discussion and cause patient anxiety [35]; relationships within the MDT are often longstanding with pre existing hierarchies which can present barriers to new user integration[41]Nevertheless, if patients are to be included in MDT meetings, clarity is required on how patients, their supporters and healthcare teams are supported to make it a positive and worthwhile experience[42].

Of key importance is that the MDT meeting is not a discussion of which option is 'best' for a particular patient, but should instead aim to determine which valid treatment options are available. In particular, palliative options (or options of 'doing nothing') are often inadequately explored [43]. Clinic structures should be flexible to allow patients to distribute their decision-making amongst information sources and people. The patient may be enabled to come to the initial consultation more informed and prepared for the discussion. There may be a role for pre MDT clinic with the patient meeting a surgeon, oncologist or specialist nurse, or a post MDT clinic to convey options and explore values and preferences, maybe with more than one clinician. The MDT meeting may take place in a small 'combined clinic' setting around the interaction with the patient. The MDT members provide support, resources and personnel to discuss the treatment options, communicate the risk and uncertainty, elicit values and explore them; a decision aid may support this work [44]. The team may consider providing an individual who is independent of the clinical team to act as a decision coach or navigator [45]. MDT members should be encouraged to update their training in supporting patients in shared decision making, consent and communication. This study provides a novel and rich account of the difficulties that patients face when making a decision in the context of

For peer review only - http://bmjopen.bmj.com/site/about/guidelines.xhtml

an MDT. Sampling included patients with a decision to make or options available, which potentially excluded more straightforward cases which may make up a lot of MDT workload. MDT decision making is mandated internationally however the specific structure of the decision process varies widely. Although the structure presented here (MDT meeting without a patient present, recommendation delivered to the patient separately) is common, other models of MDT decision making may not face similar challenges. Also, ethnographic methods, in providing depth to explore a smaller number of concepts in more detail, may lack the breadth of findings to make this piece of work widely applicable. Nevertheless, whilst the setting may not be universally generalisable, we hope that the emergent conclusions will be.

It is time for the development and design of alternative models of team decision making which have a central role for the patient. Further work to develop new model of delivering team decision-making would be multifactorial, incorporating the development of the structure of the MDT meeting and clinic, support and training for MDT members and patients and the development of tools to be used in combination with team decisions. Qualitative approaches should explore stakeholders' views of intervention components, which should be co-designed with patients. Evaluation of such interventions requires novel trial design, comparing methods of decision making and evaluating decision quality. MDT decision making is now ubiquitous and therefore the urgent need of reform to meet the principles of shared decision making should be a priority for clinical teams and cancer researchers.

Author Contributions

DW Hamilton
Protocol development, ethical approval, data gathering, data analysis, manuscript preparation

B Heaven
Protocol development, ethical approval, data analysis, manuscript preparation

R G Thomson
Protocol development, data analysis, manuscript preparation

For peer review only - http://bmjopen.bmj.com/site/about/guidelines.xhtml

JA Wilson
Protocol development, data analysis, manuscript preparation

C Exley
Protocol development, ethical approval, data analysis, manuscript preparation

Competing interests

All authors have completed the Unified Competing Interest form at www.icmje.org/coi_disclosure.pdf (available on request from the corresponding author) and declare that DWH, BH, RT, JAW and CE have no relationships with any companies that might have an interest in the submitted work in the previous 3 years; and DWH, BH, RT, JAW and CE have no non-financial interests that may be relevant to the submitted work

Funding

This study represents independent research funded by the National Institute of Health Research. The views expressed are those of the author(s) and not necessarily those of the National Health Service, the National Institute of Health Research or the Department of Health DRF-2010-03-54.

Data sharing statement

No additional data available

Ethics statement

Ethical approval was gained from the NHS Research Ethics Newcastle and North Tyneside 2 committee (reference 11/NE/0200) in September 2011 and all necessary local Research and Development governance permissions were obtained.

References

1. Licitra L, Keilholz U, Tahara M, et al. Evaluation of the benefit and use of multidisciplinary teams in the treatment of head and neck cancer. *Oral Oncol* 2016;59:73-9. doi: 10.1016/j.oraloncology.2016.06.002

2. Calman KC, Hine D. A Policy Framework for Commissioning Cancer Services In: Health Do, ed., 1995.

3. Kelly SL, Jackson JE, Hickey BE, et al. Multidisciplinary clinic care improves adherence to best practice in head and neck cancer. *American journal of otolaryngology* 2013;34(1):57-60. doi: 10.1016/j.amjoto.2012.08.010

For peer review only - http://bmjopen.bmj.com/site/about/guidelines.xhtml

4. Patil RD, Meinzen-Derr JK, Hendricks BL, et al. Improving access and timeliness of care for veterans with head and neck squamous cell carcinoma: A multidisciplinary team's approach. *Laryngoscope* 2016;126(3):627-31. doi: 10.1002/lary.25528

5. Starmer H, Sanguineti G, Marur S, et al. Multidisciplinary head and neck cancer clinic and adherence with speech pathology. *Laryngoscope* 2011;121(10):2131-5. doi: 10.1002/lary.21746

6. Taylor C, Munro AJ, Glynne-Jones R, et al. Multidisciplinary team working in cancer: what is the evidence? *Bmj* 2010;340:c951. doi: 10.1136/bmj.c951

7. NCAT. Multidisciplinary team members' views about MDT working: results from a survey commissioned by the National Cancer Action Team. London Kings College, 2009.

8. Rajan S, Foreman J, Wallis MG, et al. Multidisciplinary decisions in breast cancer: does the patient receive what the team has recommended? *British journal of cancer* 2013;108(12):2442-7. doi: 10.1038/bjc.2013.267

9. Blazeby JM, Wilson L, Metcalfe C, et al. Analysis of clinical decision-making in multi-disciplinary cancer teams. *Annals of oncology : official journal of the European Society for Medical Oncology / ESMO* 2006;17(3):457-60. doi: 10.1093/annonc/mdj102

10. Wood JJ, Metcalfe C, Paes A, et al. An evaluation of treatment decisions at a colorectal cancer multi-disciplinary team. *Colorectal disease : the official journal of the Association of Coloproctology of Great Britain and Ireland* 2008;10(8):769-72. doi: 10.1111/j.1463-1318.2007.01464.x

11. Taylor C, Atkins L, Richardson A, et al. Measuring the quality of MDT working: an observational approach. *BMC cancer* 2012;12:202. doi: 10.1186/1471-2407-12-202

12. Rowlands S, Callen J. A qualitative analysis of communication between members of a hospital-based multidisciplinary lung cancer team. *Eur J Cancer Care* 2013;22(1):20-31. doi: 10.1111/ecc.12004

13. Lanceley A, Savage J, Menon U, et al. Influences on multidisciplinary team decision-making. *Int J Gynecol Cancer* 2008;18(2):215-22. doi: DOI 10.1111/j.1525-1438.2007.00991.x

14. Soukup T, Murtagh G, Lamb BW, et al. Degrees of Multidisciplinarity Underpinning Care Planning for Patients with Cancer in Weekly Multidisciplinary Team Meetings: Conversation Analysis. *J Multidiscip Healthc* 2021;14:411-24. doi: 10.2147/JMDH.S270394 [published Online First: 2021/02/27]

15. Kidger J, Murdoch J, Donovan JL, et al. Clinical decision-making in a multidisciplinary gynaecological cancer team: a qualitative study. *Bjog-Int J Obstet Gy* 2009;116(4):511-17. doi: DOI 10.1111/j.1471-0528.2008.02066.x

16. Horlait M, Baes S, Dhaene S, et al. How multidisciplinary are multidisciplinary team meetings in cancer care? An observational study in oncology departments in Flanders,

For peer review only

For peer review only - http://bmjopen.bmj.com/site/about/guidelines.xhtml

Belgium. *J Multidiscip Healthc* 2019;12:159-67. doi: 10.2147/JMDH.S196660 [published Online First: 2019/03/14]

17. Horlait M, De Regge M, Baes S, et al. Exploring non-physician care professionals' roles in cancer multidisciplinary team meetings: A qualitative study. *PLoS One* 2022;17(2):e0263611. doi: 10.1371/journal.pone.0263611 [published Online First: 2022/02/04]

18. Hamilton DW, Heaven B, Thomson RG, et al. Multidisciplinary team decision-making in cancer and the absent patient: a qualitative study. *BMJ Open* 2016;6(7):e012559. doi: 10.1136/bmjopen-2016-012559 [published Online First: 2016/07/23]

19. Taylor C, Finnegan-John J, Green JS. "No decision about me without me" in the context of cancer multidisciplinary team meetings: a qualitative interview study. *BMC health services research* 2014;14:488. doi: 10.1186/s12913-014-0488-2

20. Strauss AL. Continual permutations of action. New York: Aldine de Gruyter 1993.

21. Charmaz K, editor. *Constructing Grounded Theory. A Practical Guide Through Qualitative Analysis*. London: SAGE, 2006.

22. Strauss AL, Corbin JM. Basics of qualitative research : grounded theory procedures and techniques. Newbury Park, Calif.: Sage Publications 1990.

23. Dey I. Grounding grounded theory : guidelines for qualitative inquiry. San Diego: Academic Press 1999.

24. Green J, Thorogood N. Qualitative methods for health research. 2nd ed. Los Angeles: SAGE 2009.

25. Elwyn G, Durand MA, Song J, et al. A three-talk model for shared decision making: multistage consultation process. *Bmj* 2017;359:j4891. doi: 10.1136/bmj.j4891 [published Online First: 2017/11/08]

26. NICE. Improving Outcomes in Head and Neck Cancer - the Manual. London, 2004.

27. Maharaj AD, Evans SM, Zalcberg JR, et al. Barriers and enablers to the implementation of multidisciplinary team meetings: a qualitative study using the theoretical domains framework. *BMJ quality & safety* 2021;30(10):792-803. doi: 10.1136/bmjqs-2020-011793 [published Online First: 2020/11/29]

28. Charles C, Gafni A, Whelan T. Decision-making in the physician-patient encounter: revisiting the shared treatment decision-making model. *Social science & medicine* 1999;49(5):651-61. doi: S0277953699001458 [pii] [published Online First: 1999/08/19]

29. Hamilton DW, Bins JE, McMeekin P, et al. Quantity vs quality of life in laryngeal cancer: a time trade off study. *Clinical Otolarygology* 2012;37(Suppl. 1):108.

30. Hamilton DW, Pedersen A, Blanchford H, et al. A comparison of attitudes to laryngeal cancer treatment outcomes: A time trade-off study. *Clinical Otolaryngology* 2018;43(1):117-23. doi: 10.1111/coa.12906

For peer review only - http://bmjopen.bmj.com/site/about/guidelines.xhtml

31. Slevin ML, Stubbs L, Plant HJ, et al. Attitudes to chemotherapy: comparing views of patients with cancer with those of doctors, nurses, and general public. *Bmj* 1990;300(6737):1458-60. [published Online First: 1990/06/02]

32. Rapley T. Distributed decision making: the anatomy of decisions-in-action. *Sociol Health Illn* 2008;30(3):429-44. doi: SHIL1064 [pii]

10.1111/j.1467-9566.2007.01064.x [published Online First: 2008/01/16]

33. Entwistle VA, Carter SM, Cribb A, et al. Supporting patient autonomy: the importance of clinician-patient relationships. *J Gen Intern Med* 2010;25(7):741-5. doi: 10.1007/s11606-010-1292-2

34. Diekmann A, Heuser C, Ernstmann N, et al. How do breast cancer patients experience multidisciplinary tumor conferences? - A description from the patient perspective. *Breast* 2019;44:66-72. doi: 10.1016/j.breast.2018.12.012 [published Online First: 2019/01/23]

35. Butow P, Harrison JD, Choy ET, et al. Health professional and consumer views on involving breast cancer patients in the multidisciplinary discussion of their disease and treatment plan. *Cancer* 2007;110(9):1937-44. doi: 10.1002/cncr.23007

36. Heuser C, Diekmann A, Schellenberger B, et al. Patient Participation in Multidisciplinary Tumor Conferences from the Providers' Perspective: Is It Feasible in Routine Cancer Care? *J Multidiscip Healthc* 2020;13:1729-39. doi: 10.2147/JMDH.S283166 [published Online First: 2020/12/05]

37. Bellardita L, Donegani S, Spatuzzi AL, et al. Multidisciplinary Versus One-on-One Setting: A Qualitative Study of Clinicians' Perceptions of Their Relationship With Patients With Prostate Cancer. *Journal of oncology practice / American Society of Clinical Oncology* 2011;7(1):e1-5. doi: 10.1200/JOP.2010.000020

38. Choy ET, Chiu A, Butow P, et al. A pilot study to evaluate the impact of involving breast cancer patients in the multidisciplinary discussion of their disease and treatment plan. *Breast* 2007;16(2):178-89. doi: 10.1016/j.breast.2006.10.002

39. Bohmeier B, Schellenberger B, Diekmann A, et al. Opportunities and limitations of shared decision making in multidisciplinary tumor conferences with patient participation - A qualitative interview study with providers. *Patient education and counseling* 2021;104(4):792-99. doi: 10.1016/j.pec.2020.09.007 [published Online First: 2020/10/15]

40. Heuser C, Diekmann A, Kowalski C, et al. Health literacy and patient participation in multidisciplinary tumor conferences in breast cancer care: a multilevel modeling approach. *BMC cancer* 2019;19(1):330. doi: 10.1186/s12885-019-5546-z [published Online First: 2019/04/10]

41. Martin GP, Finn R. Patients as team members: opportunities, challenges and paradoxes of including patients in multi-professional healthcare teams. *Sociol Health Illn* 2011;33(7):1050-65. doi: 10.1111/j.1467-9566.2011.01356.x

For peer review only - http://bmjopen.bmj.com/site/about/guidelines.xhtml

42. Schellenberger B, Heuser C, Diekmann A, et al. Questions and emotional expressions from patients and companions while participating in multidisciplinary tumor conferences in breast and gynecological cancer centers. *Patient education and counseling* 2021 doi: 10.1016/j.pec.2021.12.010 [published Online First: 2021/12/27]

43. Nelson A, Longo M, Byrne A, et al. Chemotherapy decision-making in advanced lung cancer: a prospective qualitative study. *BMJ Support Palliat Care* 2020 doi: 10.1136/bmjspcare-2020-002395 [published Online First: 2020/08/23]

44. Stacey D, Legare F, Lewis K, et al. Decision aids for people facing health treatment or screening decisions. *Cochrane Db Syst Rev* 2017(4) doi: ARTN CD001431

10.1002/14651858.CD001431.pub5

45. Stacey D, Murray MA, Legare F, et al. Decision coaching to support shared decision making: a framework, evidence, and implications for nursing practice, education, and policy. *Worldviews Evid Based Nurs* 2008;5(1):25-35. doi: WVN108 [pii]

10.1111/j.1741-6787.2007.00108.x [published Online First: 2008/02/13]

For peer review only

Staff Interview Schedule

Introduce researcher and purpose of the study

Obtain consent to proceed and to record the conversation

- What do you see as the best way of making decisions in head and neck cancer?
- The MDT
  - What is it for? What is its primary aim?
  - What works well, and what works not so well?
  - How could it be improved?
  - No decision about me without me in the MDT…. Is this possible?
- Treatment decision
  - Why do you regard a treatment as the best for a patient?
  - What factors do you take into account when making treatment decisions?
- Patient involvement in decisions/shared decision making
  - What does it mean to you?
  - Do you think there are barriers to patient involvement? What are they? Why do they exist?
  - Why do clinicians struggle to involve their patients effectively?
  - Do you think we need to improve patient involvement? Why?
- Uncertainty/conflict
  - Is it a problem or is it healthy/required?
  - How should uncertainty and conflict be presented to the patient? Should they know that you don't know?
  - If there are options, how should they be communicated?
- Decision for treatment
  - What is the role of the patient in the treatment decision?
  - How much of a role should the patient have? Can patients know enough?
  - How much of a role should a patient have in the decision
    - Should we give power of vito?
    - Should you allow a patient to make a decision which is considered wrong?
    - how much should a patient know before treatment
  - How much of a role should the family have
- Palliation/prognosis
  - Do we palliate enough? Do we treat too many people radically?
  - What are the barriers to good palliation?
  - Do you think we have a range of palliative options available to us?
  - Do you have prognostic information available to you?
  - Would you use this information if it was available? How would you use it?

For peer review only

New patient interview 1 schedule

Introduce researcher and purpose of the study

Obtain consent to proceed and to record the conversation

The interview will cover the following broad areas:

- Previous experience of making healthcare decisions
  - What does shared decision making mean?
  - Have you had to make big healthcare decisions before?
- Experience of making this decision
  - What was your process of making the decision? Talk me though it
  - What did you draw upon?
  - What factors did you take into account?
  - Did you turn to anybody/anything for information or help?
  - How did you come to your final decision?
  - What was the MDT like?
  - Did you understand what was going on?
  - Could it be improved?
- Information given about the disease and treatment options
  - Do you think you had enough information?
  - Where do you get your information from?
  - Do you think you need more information?
  - Did you understand all the information
- Patient involvement in the decision about care
  - Should patients be involved in decisions about their care?
  - How should we involve patients?
  - Were you involved? How?
  - Would you have liked more say/less say?
  - How this made the participant feel, and further exploration
- What are your expectations of treatment?
  - Are you expecting side effects? What sort?
  - Do you have any idea of your prognosis? Do you want to know?
  - Where will you be in 6 months time?
- In the perfect world, how do you think decisions should be made?

For peer review only

New patient interview 2 schedule/retrospective patient interview schedule

Introduce researcher and purpose of the study

Obtain consent to proceed and to record the conversation

- Experience of the treatment
    - What has happened so far, story of the treatment
    - Correlation with expectations – worse, better
- New normal
    - Do you have a good quality of life?
    - How do you cope with swallowing?
    - How is your voice, how do you get on with communication?
- MDT and decision process
    - When you look back did you understand the treatment decision?
    - Did you understand the consequences of the decision?
    - Did you have enough information to base the decision on?
    - Should they have allowed you more or less control over the decision?
    - Do you wish the decision had been made differently?
    - Do you feel you made the right or the wrong decision?
    - Do you have any regrets?
    - How could they improve the clinic/the decision process?
    - In the perfect world, how do you think treatment decisions should be made?
- Palliation/prognosis
    - Were you ever aware of the chances of the treatment being successful?
    - Would you want to be aware?
    - Was palliation ever an option for you? Is it now?
- Information giving
    - Do you think you had enough information about treatment?
    - Did you understand what you were entering yourself into?
    - Should they give more information? Could they?

For peer review only

Topic	Page	Short description
1: Title	1	How do patients make decisions in the context of a multidisciplinary team: an ethnographic study of four head and neck cancer centres
2: Abstract	2-3	Included in manuscript
3: Problem formulation	4-5	MDT working is common and expensive, but the impact on shared decision making and patient involvement in decisions remains relatively unexplored
4: Purpose or research question	4-5	To examine critically patient engagement in the MDT treatment decision making process in head and neck cancer, and to evaluate the experience and practice of decision-making by patients and clinicians
5: Qualitative approach and research paradigm	5-7	Ethnographic methods (non-participant observation and semi-structured interviews). Analysis followed principles of constructivist grounded theory
6 Researcher characteristics and reflexivity	5	All data were collected by the lead author (DWH) whilst performing his PhD. At the time, he was a trainee surgeon and had taken time out of programme for research. The dual role of surgical trainee and researcher allowed easy access to the setting and language, making ethnography and observations easier and potentially more 'natural'. Co-authors BH and CE were involved in analysis in order to minimise the effect of the researcher's status on the emerging conclusions
7 Context	6&7	The research was conducted in four head and neck cancer centres in the north east of England. In all centres, the MDT meeting took place without the patient present; following this, one or more members of the MDT met with the patient in clinic
8 Sampling strategy	7	Initial sampling aimed to recruit patients who had a treatment decision to make about their care, or where more than one treatment option was available to the patient. . Purposive sampling guided the sampling strategy to explore emerging concepts with data collection and analysis occurring in tandem. Thus, further sampling was guided by the emerging analysis and continued until a state of theoretical sufficiency was achieved.
9 Ethical issues pertaining to human subjects	5	Ethical approval was gained from the NHS Research Ethics Newcastle and North Tyneside 2 committee (reference 11/NE/0200) in September 2011 and all necessary local Research and Development governance permissions were obtained. All participants provided informed consent.
10 Data collection methods	6	Non-participant observation; semi structured interviews with patients and staff; field notes; reflective notes. Iterative process with emerging themes explored through further data collection. All

For peer review only

		emerging analysis and memos discussed with co-authors (CE and BH)
11 Data collection instruments and technologies	6	Interview guides (iteratively developed) used for interviews. Field notes and notes from informal discussions. Audiorecording and word-for-word transcription of all formal interviews, MDT meeting and MDT clinic
12 Units of study	6	34 observations of MDT meetings and 37 clinic appointments (for 30 patients; 13 of these patients were interviewed once, three were interviewed twice). Interviews with four further treated patients and nine members of staff.
13 Data processing	6-7	All audiorecordings were transcribed word for word and anonymised. All reflective notes and field notes were anonymised. Patients and staff given pseudonyms. MDT meeting data transcribed by the main author (DWH) and all recording transcribed by others were checked by DWH. Audio recordings destroyed after transcription. Data kept on University computers, password protected
14 Data analysis	7	The data were analysed by one researcher (DWH) and emerging analyses were discussed with CE and BH, following principles of constructivist grounded theory. All data sources (MDT meeting, clinic, informal and formal interviews) were analysed using the same coding framework. Line by line coding produced an initial coding framework: the emerging analysis was used during axial coding to guide further sampling and further development of the coding framework; when the coding framework was altered, all transcripts were re-coded. The codes used were conceptual, rather than descriptive, and labels were derived completely from the data, not pre-determined. The coding was organised using the NVivo computer package. Emerging findings (and 'memos') were discussed in the research team (particularly with CE and BH) to develop the data analysis and guide subsequent analysis and data sampling
15 Techniques to enhance trustworthiness	7	All emerging themes and analysis were discussed in the research team with CE and BH involved in analysis. Findings presented in departmental meetings
16 Synthesis and interpretation	7-12	The outcome of an MDT discussion is frequently a "in the clinic as they confront a terrifying diagnosis and a myriad of complex decision options. In turn,.
17 Links to empirical data	7-12	Data presented in the results section linked to empirical data throughout
18 Integration with prior work, implications, transferability and contributions to the field	12-16	The discussion section discusses the findings in the context of prior work and the contributions to the field

For peer review only

19 Limitations	3, 18-19	Strengths and limitations are discussed in the discussion section and there is a list of strengths and limitations at the beginning of the manuscript
20 Conflict of interest	16	All authors have completed the Unified Competing Interest form at www.icmje.org/coi_disclosure.pdf (available on request from the corresponding author) and declare that DWH, BH, RT, JAW and CE have no relationships with any companies that might have an interest in the submitted work in the previous 3 years; and DWH, BH, RT, JAW and CE have no non-financial interests that may be relevant to the submitted work
21 Funding	17	This study represents independent research funded by the National Institute of Health Research. The views expressed are those of the author(s) and not necessarily those of the National Health Service, the National Institute of Health Research or the Department of Health

For peer review only
